# Protein and Peptide Composition of Male Accessory Glands of *Apis mellifera* Drones Investigated by Mass Spectrometry

**DOI:** 10.1371/journal.pone.0125068

**Published:** 2015-05-08

**Authors:** Vladimir Gorshkov, Wolfgang Blenau, Gudrun Koeniger, Andreas Römpp, Andreas Vilcinskas, Bernhard Spengler

**Affiliations:** 1 Institute of Inorganic and Analytical Chemistry, Justus Liebig University, Giessen, Germany; 2 Zoological Institute, University of Cologne, Cologne, Germany; 3 Bee Research Institute, Goethe University, Frankfurt, Germany; 4 Institute of Phytopathology and Applied Zoology, Justus Liebig University, Giessen, Germany; San Diego, UNITED STATES

## Abstract

In honeybees, reproductive females usually mate early in their life with more than 10 males in free flight, often within 10 minutes, and then store male gametes for up to five years. Because of the extreme polyandry and mating in free flight special adaptations in males are most likely. We present here the results of an investigation of the protein content of four types of male reproductive glands from the Western honeybee (*Apis mellifera*) drone, namely seminal vesicles (secretion in ejaculate), as well as bulbus, cornua and mucus glands (secretions for the mating plug). Using high resolution and accuracy mass spectrometry and a combination of database searching and *de novo* sequencing techniques it was possible to identify 50 different proteins in total, inside all mentioned glands, except in the mucus gland. Most of the proteins are unique for a specific gland type, only one of them (H9KEY1/ATP synthase subunit O) was found in three glands, and 7 proteins were found in two types of glands. The identified proteins represent a wide variety of biological functions and can be assigned to several physiological classes, such as protection, energy generation, maintaining optimal conditions, associated mainly with vesicula seminalis; signaling, cuticle proteins, icarpin and apolipoproteins located mainly in the bulbus and cornua glands; and some other classes. Most of the discovered proteins were not found earlier during investigation of semen, seminal fluid and tissue of reproductive glands of the bee drone. Moreover, we provide here the origin of each protein. Thus, the presented data might shed light on the role of each reproductive gland.

## Introduction

The ability to store significant amounts of male gametes for a long period of time (several years) is a characteristic feature of the queens of social Hymenoptera [1]. The queens of the honeybee (*Apis mellifera*) mate only once, early in their adult life, and accumulate the amount of sperm sufficient for the complete lifetime [2–4]. Sperm storage by female insects is an important feature of reproductive biology, as it temporally separates mating from egg fertilization. It potentially increases fecundity as more eggs can mature over time and be fertilized with sperm from a previous mating [1]. Sperm storage is an active process involving the steps of attracting sperm to the site of storage, keeping them alive, and activating them for fertilization [5]. It is believed that both male and female contribute to the sperm storage process [6]. Male ejaculate consists of sperm itself and seminal fluid or seminal plasma, with the latter being crucial for fertility and post-copulatory sexual selection [7]. The components of seminal fluid have a wide variety of biological functions, for example they are involved in retaining sperm viability, protecting it from microbial attacks and oxidative stress [6,8–12]. In some insects, the components of seminal fluids are also known to influence female physiology and behavior, including changing female likelihood of re-mating, increasing ovulation and egg-laying rates, changing female flight and feeding habits etc. [9,13]. In case of social Hymenoptera, the supportive function of seminal fluid is even more important, since the size and longevity of the colony is dependent on the quality of gametes stored by the queen [14]. In honeybees, queens actively participate in sperm maintenance and these female contributions are likely to be of central importance for sperm survival. The spermathecal fluid proteins of the honeybee differ substantially from those in the seminal fluid [15] supporting the idea that selection on seminal and spermathecal fluid are substantially different. Seminal fluid was selected to increase insemination and paternity success whereas spermathecal fluid evolved to maximize sperm survival [15].

**Fig 1:**
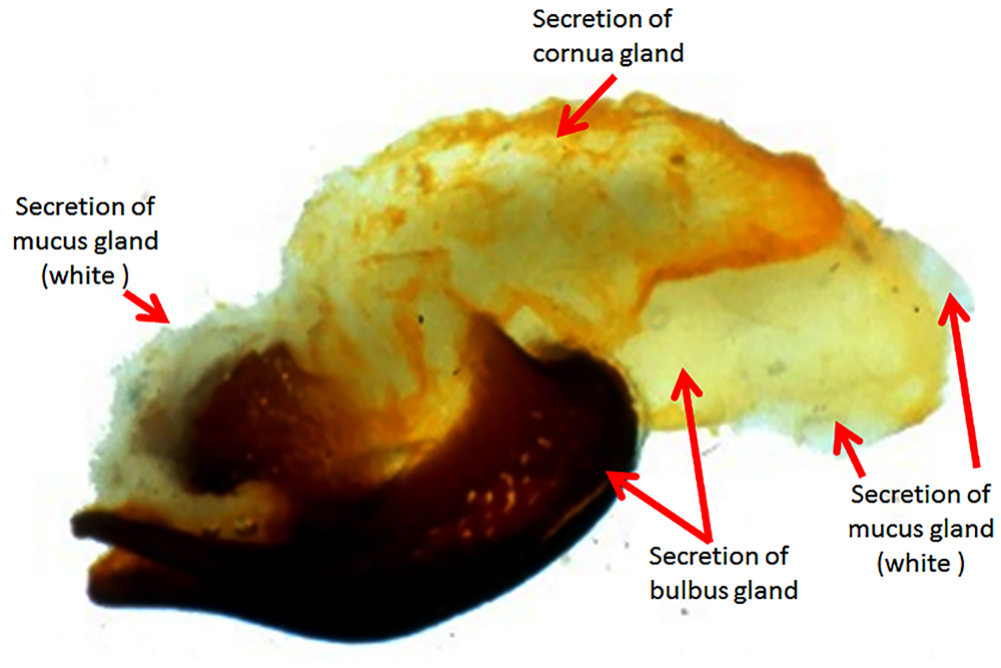
Structure of the "mating sign."

In insects, the male accessory glands are generally assumed to be major contributors towards seminal fluid. Therefore, their molecular make up and physiology has attracted much attention [10]. The reproductive system of bee drone consists of several types of organs, the most important of which are paired testes and seminal vesicles forming the ejaculate, mucus, bulbus and cornua glands [16–18]. During the mating process, the huge membranous penis (endophallus) is everted. Through the constriction of seminal vesicles the sperm is transferred into the queen’s vaginal passage and oviducts. The secretion of mucus glands enveloped by the bulbus gland secretion follows the semen but remains in the sting chamber and hardens on the contact with air, forming a mating plug [19,20]. Finally the mainly lipid containing secretion of the cornua gland is added. After the eversion, the male separates by pulling out the endophallus leaving coagulating mucus and yellowish secretions of the bulbus and cornua glands attached to the queen as so-called “mating sign” (Fig 1) [21,22]. Mucus and genital structures are considered to help fixing the male in place during the process of sperm transfer [22]. The mating sign, which is easily removed by the next drone and thus cannot physically prevent the queen from mating with other drones, was originally thought to prevent backflow of semen immediately after mating but is now interpreted as “advertising a high quality mated queen” to other drones competing in a drone congregation area [23]. Moreover it might also protect the male endophallus from the queen’s sting [24] and may contribute to guarantee sterile conditions during sperm transfer of up to 20 drone bees or even more [25].

The major part of honeybee drone reproductive secretion is proteins except for the cornua gland secretion containing a high quantity of lipids [26]. The protein estimate content for luminal mucus is 50–60 μg per gland pair [27]. In the best-studied species, *Drosophila melanogaster*, more than 100 accessory gland proteins were characterized so far [28], including the biological activity for some of them [9]. It was shown that proteins produced by bee drones contribute significantly to the physiology of sperm preservation and storage [6,21]. However, the relationship between protein concentration and sperm viability is not linear, which might argue in favor of the proteins present in seminal fluid having not only enzymatic functions. Moreover, the addition of cellulose or bovine serum albumin, which are not present in native seminal plasma, to inactive sperm has a similar positive effect as adding seminal plasma proteins. However, this effect is by far smaller when the sperm is activated [10]. In total, about a hundred proteins are identified in honeybee seminal fluid so far [21,29,30]. They can be distributed in several functional groups: signaling and defense, e.g. antioxidant, metal-binding, pheromone-binding proteins; energy production, e.g. amino acid synthetases, ATP or NADH synthases; protein structure/function proteins, e.g. proteases and heat shock proteins; several representatives of lipid metabolism proteins, phosphate catabolism proteins and transport proteins were also found. Apart from functional classes, proteins found can be attributed to three physiological categories: proteins involved in maintaining environment for sperm survival (energy, phosphate-binding and protein folding), proteins providing sperm with its physiological needs (energy, protection from dangerous substances) and proteins influencing female behavior [29,30].

Our goal was to investigate the protein and peptide content of four different reproduction-related glands of the honeybee drone separately in order to identify functional classes and the morphological origin of proteins synthesized by the glands. We employed database searching methods, using all bee proteins reported so far in public protein databases, as well as *de novo* sequencing, to complement database search results. Both approaches were based on high accuracy and resolution mass spectrometry (MS) measurements, allowing precise structure determination.

## Experimental

### Gland Preparation

Predominantly mature drones were collected from a hive of Western honeybees (*Apis mellifera carnica*) kept at the apiary of the Oberursel Bee Research Institute. From each drone, the reproductive apparatus was dissected and each gland was separated and rinsed three times in phosphate buffered saline. Tissue samples were stored at -20°C until further processing.

### Extraction of Gland Content

Two glands of each type were carefully mixed with 30 μL 0.1% TFA avoiding the destruction of gland tissue and sonicated for 5 min. The solution was left overnight at 4°C to complete the extraction of gland contents. Later, the samples were purified by ZipTip C18 using standard protocol, completely dried in SpeedVac and reconstituted in 1% formic acid (FA) before HPLC-MS analysis. No enzymatic digestion was performed before LC-MS analysis.

### Tandem Mass Spectrometry

All experiments were done on a 7-Tesla Finnigan LTQ-FT Ultra mass spectrometer (Thermo Fisher Scientific GmbH, Bremen, Germany) consisting of a linear quadrupole ion trap and a Fourier transform ion cyclotron resonance (FTICR) mass spectrometer, equipped with a nano electrospray ionization source run in positive ion mode (spray voltage 2.2 kV, ion transfer tube temperature 275°C). The HPLC system consisted of a solvent degasser, a nano flow pump and an autosampler (Ultimate, Dionex/LCPackings, Idstein, Germany). Chromatographic separation of the peptides took place in a 15-cm analytical column with an internal diameter of 75 μm filled with C18, 3 μm, 100 Å stationary phase. Samples were pre-focused on the trap column (Dionex, C18 PepMap, i.d. 300 μm, length 5 mm) and eluted by the following multistep gradient: 4–40% B in 7 min, 40–95% B in 35 min, and isocratic 95% B for 5 min. Solvent A was 2% acetonitrile/water with 0.1% FA, solvent B–80% acetonitrile/water with 0.1% FA. The mass spectrometer was operated in the data-dependent mode, fragmenting three most intense ions by CID (isolation width 2 u, normalized collision energy 30). Survey MS spectra (from *m*/*z* 200–2000) were acquired in the FTICR cell with a mass resolution of 100,000 (@m/z 400), while MS^2^ spectra were recorded with a mass resolution of 50,000 (@m/z 400). The mass spectrometry data have been deposited to the ProteomeXchange Consortium [31] via the PRIDE partner repository with the dataset identifier PXD001993 and 10.6019/PXD001993.

### Amino Acid Sequence Analysis

First, LC-MS data were processed by Thermo Proteome Discoverer version 1.3 (Thermo Fisher Scientific GmbH, Bremen, Germany) using the SEQUEST search engine [32] embedded in this software. The reference database included proteins of the genus *Apis* obtained from Uniprot as well as frequently observed contaminations (cRAP set: http://www.thegpm.org/crap/index.html dated 29.02.2012). Unspecific cleavages were used to form theoretical peptides; oxidation of methionine was specified as variable modification. Fragmentation spectra were grouped with 4 ppm tolerance for parent ion and 1 min RT window prior to search. The same mass tolerance was used for parent ion in SEQUEST, while for the fragments the tolerance was set to 0.01 Da. Database search results were filtered to FDR < 0.05 (estimated by target-decoy approach) and further validated by manual *de novo* sequencing. In the second step, manual *de novo* interpretation of all spectra was performed to find peptides undiscovered by SEQUEST. Two home-built programs, written in Python 2.7.5, were used to assist *de novo* sequencing. Except when standard Python packages Pyteomics [33] was used, the GUI was built using the PyQt4 (v. 4.9.6) package. The first program (Mass2aa) was used to fit the mass provided by the user, to an amino acid composition, consisting of the 20 standard amino acids including several most common modifications. The mass can be accounted as complete peptide with free acid or amide C-terminus, b- or y- ion, or as bare amino acid combination. The second program (FuzzyMatch) was employed to match incomplete sequencing results to a database of proteins. The software is capable to operate with permutation blocks, e.g. when the position of amino acids in the sequence is not definite. When a large part of sequence is unclear, it can account for small changes in the sequence, such as amino acid substitution or modification in N- or C-terminal part. Detailed description is provided in S1 Text. The same protein database as for SEQUEST search was used for matching *de novo* sequencing results.

## Results and Discussion

On the first step, the HPLC-MS data for each gland were analyzed with database searching algorithm (SEQUEST). As the result a number of matches were found, that were checked for correctness later. The important features taken into consideration were: (1) the completeness of rational explanations of all intense peaks in the spectrum, (2) the length of consecutive ion series, and (3) the interconnection between ion series (e.g. complementarity of ions, internal ions). An example of a spectrum poorly identified by SEQUEST and subsequently corrected using the *de novo* sequencing approach as illustrated in this manuscript, is presented in S2 Text.

The list of proteins identified by database search is provided in Table 1. Protein identification by only one peptide is also included in the table, since all peptide identifications were additionally checked for correctness manually, minimizing the number of false positive identifications. Moreover, some of the identifications receive additional support in the second step of data analysis. Although the secretion of mucus glands forms the major part of the mating sign, only low molecular mass compounds were identified in this gland, while proteins were discovered in three other gland types. In total 6 proteins (1 unique) were found in bulbus, 13 (8 unique) in cornua and 18 (16 unique) in vesicula seminalis. In total 31 proteins, with 73 corresponding peptides (2.4 peptide/protein) were found. S1 Table contains additional details on identified peptides and proteins.

**Table 1:** Proteins identified in reproduction-related glands of *Apis mellifera* drones only by SEQUEST.

Uniprot	RefSeq	BeeBase	Description	Function	Peptides	B	C	V
A5A5E4	-	GB46297-PA	Structural cuticle protein (Fragment) OS = Apis mellifera PE = 2 SV = 1	Structural molecule activity	8	X	X	
H9KBS5	NP_001257759	GB52161-PA	PREDICTED: flexible cuticle protein 12 OS = Apis mellifera GN = CPR28 PE = 4 SV = 1	Structural molecule activity	5	X	X	
H9KGM8	-	GB46297-PA	PREDICTED: endocuticle structural glycoprotein SgAbd-2-like OS = Apis mellifera	Structural molecule activity	17	X	X	
H9K0P8	XP_001120021	GB44777-PA	PREDICTED: hypothetical protein LOC725882 OS = Apis mellifera GN = LOC725882 PE = 4 SV = 1	Molybdopterin biosynthesis protein MoeB domain; Validated	1		X	
H9KU41	-	GB46312-PA	PREDICTED: endocuticle structural glycoprotein SgAbd-8 OS = Apis mellifera	Structural molecule activity	8	X	X	
Q6VQ13	NP_001010975	GB42422-PA	ADP/ATP translocase OS = Apis mellifera GN = Ant PE = 2 SV = 1	Transporter activity, cytoplasm, membrane, mitochondrial	5	X		X
H9KHD2	XP_392478	GB42526-PA	PREDICTED: malate dehydrogenase, mitochondrial-like isoform 1 OS = Apis mellifera	Carbohydrate metabolism, citric acid cycle	2			X
H9KC10	XP_001120194	GB45913-PA	PREDICTED: protein lethal(2)essential for life-like OS = Apis mellifera GN = LOC724488 PE = 3	Heat-shock protein 20 (HSP20)	1			X
H9KJ51	-	GB55598-PA	Troponin I OS = Apis mellifera PE = 4 SV = 1	Muscle constriction, part of troponin complex, binds actin	1			X
H9KA48	-	GB40759-PA	Icarpin precursor OS = Apis mellifera PE = 4 SV = 1	Venom carbohydrate-rich protein, secreted by venom duct, extracelular	1		X	
H9KMZ3	XP_625049	GB48244-PA	Cyclin-dependent kinase 6-like OS = Apis mellifera PE = 4 SV = 1	Kinase activity, phosphorylation, cell cycle regulation	1			X
H9KTW5	XP_624662	GB48904-PA	PREDICTED: glutathione S-transferase-like OS = Apis mellifera GN = LOC552283 PE = 3 SV = 1	Transferase, processing of xenobiotics, detoxification, diverse functions	1			X
H9K5H8	XP_392114	GB51551-PA	PREDICTED: myophilin OS = Apis mellifera GN = Chd64 PE = 4 SV = 1	Muscle associated	1			X
H9K6R4	XP_001121551	GB46094-PA	PREDICTED: actin, cytoplasmic 1-like, partial OS = Apis mellifera GN = LOC725739 PE = 4 SV = 1	Muscle constriction	1	X		
H9K538	XP_393297	GB42792-PA	PREDICTED: hypothetical protein LOC409805 OS = Apis mellifera GN = Mf PE = 4 SV = 1	Unknown	2			X
H9KIT9	XP_624806	GB43999-PA	PREDICTED: peroxiredoxin-5, mitochondrial OS = Apis mellifera GN = Prx5 PE = 4 SV = 1	Response to oxidative stress, cell redox homeostasis	2		X	X
B0LUE8	NP_001107670	GB55452-PA	Apolipophorin-III-like protein OS = Apis mellifera GN = A4 PE = 2 SV = 1	Lipid transport, extracelular	1		X	
H9KMZ2	-	-	PREDICTED: hypothetical protein LOC552453 OS = Apis mellifera GN = LOC552453 PE = 4 SV = 1	Concerved Domain: Protein of unknown function (DUF745); Drosophila proteins family	1			X
H9K1E2	XP_006559246	GB54446-PA	Arginine kinase OS = Apis mellifera GN = Argk PE = 3 SV = 1	Phosphorylation, arginine metabolism, proline metabolism	1			X
H9KKD2	XP_001121746	GB53748-PA	PREDICTED: hypothetical protein LOC725960 OS = Apis mellifera GN = LOC725960 PE = 4 SV = 1	Unknown	1		X	
H9K7H5	XP_001122696	GB42797-PA	PREDICTED: protein takeout-like OS = Apis mellifera GN = LOC726981 PE = 4 SV = 1	Probably: chemoreception; Probably: circadian-controlled	1		X	
H9KGE6	XP_393789	GB47268-PA	PREDICTED: electron transfer flavoprotein subunit beta-like OS = Apis mellifera	Phosphorylation, electron transport, microtubule associated, lipid particle associated	1		X	
Q5XUU6	NP_001011640	GB48492-PA	Take-out-like carrier protein JHBP-1 OS = Apis mellifera PE = 2 SV = 1	Transfer of Juvenile Hormone PFAM: Pf06585	1		X	
H9KR59	XP_006568820	GB55482-PA	PREDICTED: Na(+)/H(+) exchange regulatory cofactor NHE-RF1-like OS = Apis mellifera	PDZ signaling domain: protein-protein interaction, signaling	1			X
H9KP88	-	GB54676-PA	Aspartate aminotransferase OS = Apis mellifera GN = Ame.2149 PE = 3 SV = 1	Aminoacid metabolism, alkaloid synthesis	1			X
H9K024	XP_006567025	GB52107-PA	Tubulin alpha OS = Apis mellifera GN = LOC408388 PE = 3 SV = 1	Tubulin complex, microtubule component, protein polymeryzation, humoral response	1			X
H9K626	XP_393545	GB52073-PA	PREDICTED: probable citrate synthase 1, mitochondrial-like OS = Apis mellifera	Carbohydrate metabolism, citric acid cycle	1			X
H9KNB7	XP_003251464	GB41306-PA	Actin OS = Apis mellifera GN = LOC410075 PE = 3SV = 1	Muscle constriction	1			X
H9KPL4	XP_392899	GB54372-PA	PREDICTED: 60 kDa heat shock protein, mitochondrial-like OS = Apis mellifera	Response to stress, protein refolding, transmembrane transport, pH regulation	1			X
H9K918	XP_006564892	GB52736-PA	Probably: ATP synthase subunit beta OS = Apis mellifera GN = Atp5b PE = 3 SV = 1	Oxidative phosporylation, proton transport, ATP synthesis	1		X	
H9KR70	XP_625171	GB53437-PA	PREDICTED: f-box only protein 32-like OS = Apis mellifera PE = 4 SV = 1	Cell cycle regulation, signal transduction	1			X

Uniprot accession number, functional description, number of peptides supporting the protein and the origin are provided for each protein. B—bulbus gland, C—cornua gland, V—vesicula seminalis.

On the next step *de novo* sequencing was applied to all unidentified spectra. During this process peptides with modifications, unusual peptides and those which were missed by SEQUEST can be identified. Analysis of fragmentation spectra was performed manually. Home-built software was used to match sequencing results with the database of proteins. Several interesting examples of this analysis can be found below and in S3 Text.

**Fig 2:**
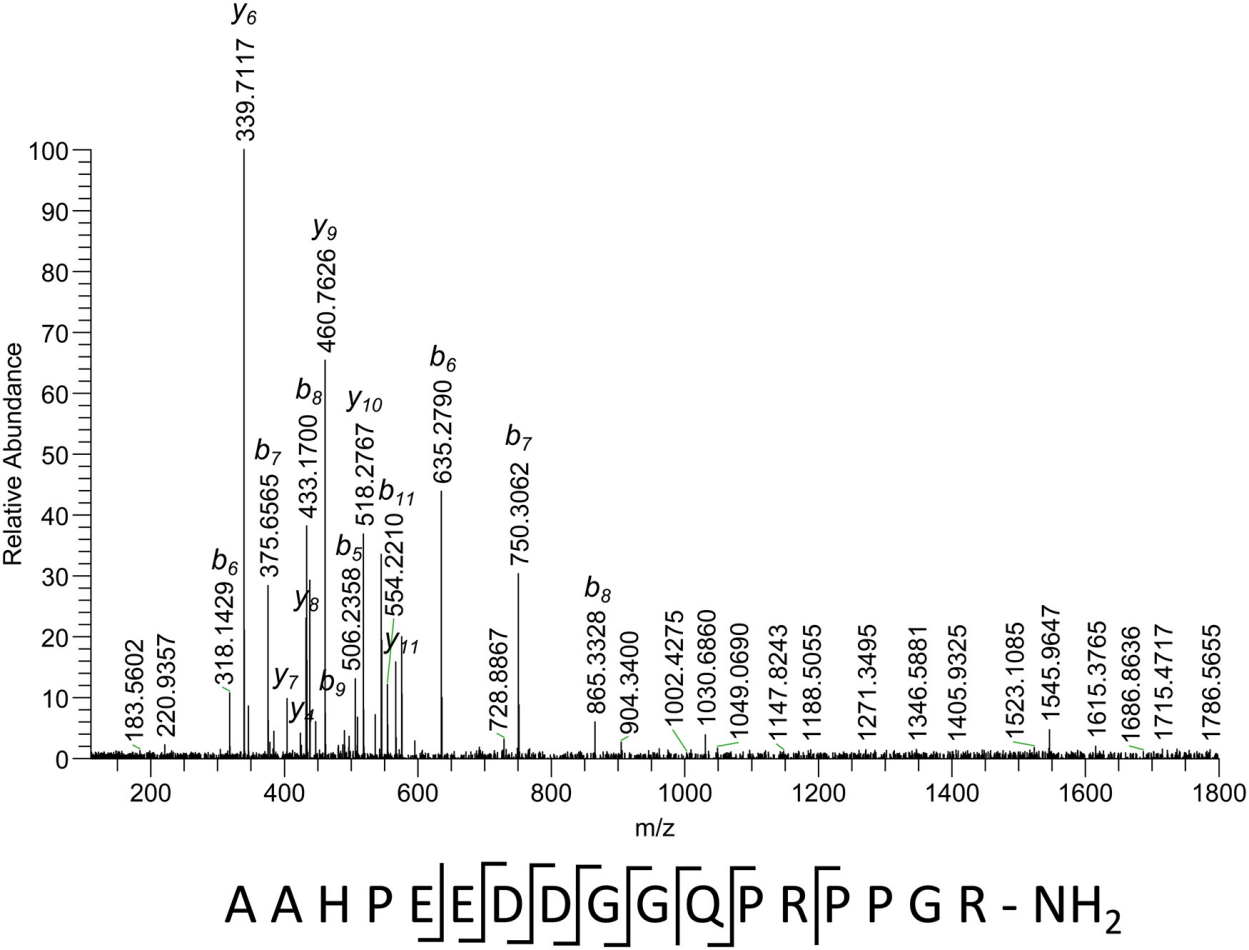
Fragmentation spectrum of a peptide with accurate measured mass of 1783.8352 Da (M), quadruply-charged precursor (m/z 446.9661). The sequence under the spectrum shows observed cleavages.

Fig 2 shows the fragmentation spectrum of the quadruply-charged peptide, having the molecular weight of 1783.8352 Da. The ions observed correspond to fragmentation in the middle of the sequence, giving the following sequence tag: (506.2359)EDDGGQ(253.1552)(425.2620). The digits in brackets represent the accurate measured masses of undisclosed parts. A mass difference of 253.1552 Da could be only connected with a proline-arginine combination with 5.23 ppm error. According to the proline rule, proline should be in front of arginine in a sequence (PR and not RP). A mass difference of 506.2359 Da has 104 different possible amino acid compositions, including C- and N- terminal ones. For mass 425.2620 Da 4 different compositions can be found. As a result we have more than 800 different possible amino acid sequences for this peptide.

**Fig 3:**
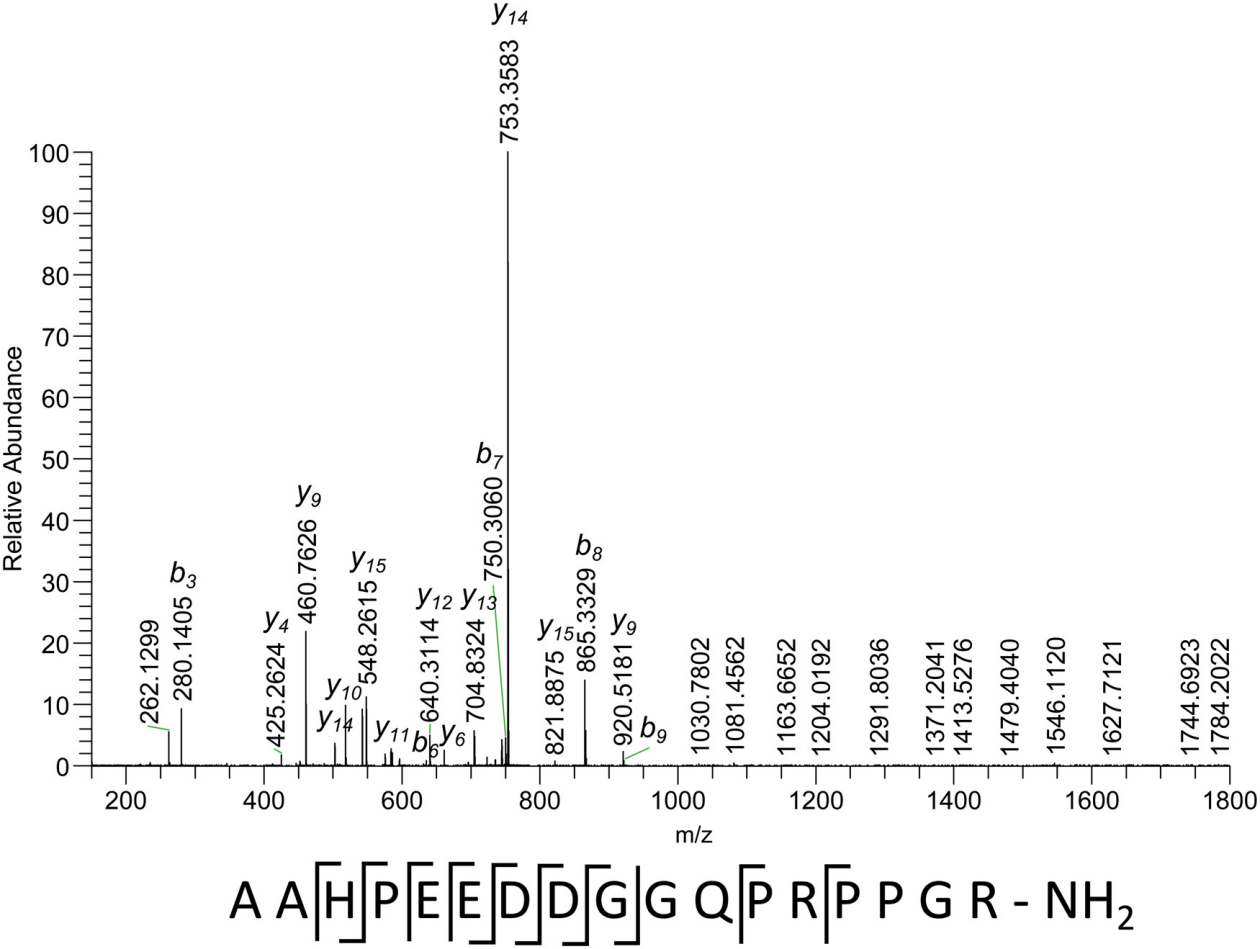
Fragmentation spectrum of a peptide with accurate measured mass of 1783.8352 Da (M), triply-charged precursor (m/z 595.6189). The sequence under the spectrum shows observed cleavages.

The fragmentation spectrum of the triply charged precursor of the same peptide (Fig 3) shows a different fragmentation pattern, most of the cleavages take place in the N-terminal part. Some of the ions are common between the two fragmentation spectra. With the help of this spectrum it is possible to find the structure of the 506 Da N-terminal gap discussed above. The only possible structure of b_2_-ion according to accurate mass is the pair of two alanine amino acids. A weak noise-level peak corresponding to the cleavage between these two amino acids (y_16_ m/z 1713.7759 and m/z 857.3939) gives this hypothesis additional support. No additional cleavages were observed in the C-terminal part. Therefore its structure remains unclear. Using the two fragmentation spectra of the same peptide, it is possible to establish the partial sequence AAHPEEDDGGQ(PR)(425.2620). Matching it against the database gave three hits with identical sequence AAHPEEDDGGQPRPPGR-OH. All three of them are related to different types of structural cuticle protein. It is worth mentioning that the sequence of the proline-arginine pair is as predicted by the proline rule. The difference in the mass of the database peptides compared to the measured one is -0.9839 Da. This observed mass difference should be due to some modification of the non-sequenced C-terminal part. Its sequence is PPGR, and the most probable explanation of the mass shift is an amidation of the C-terminus, since none of the amino acids (proline, glycine, arginine) present in the peptide sequence have any known modifications that can lead to this mass change. The accuracy of match for the C-terminal part (425.2620 Da) is 0.17 ppm, and for the full peptide is −0.12 ppm.

**Fig 4:**
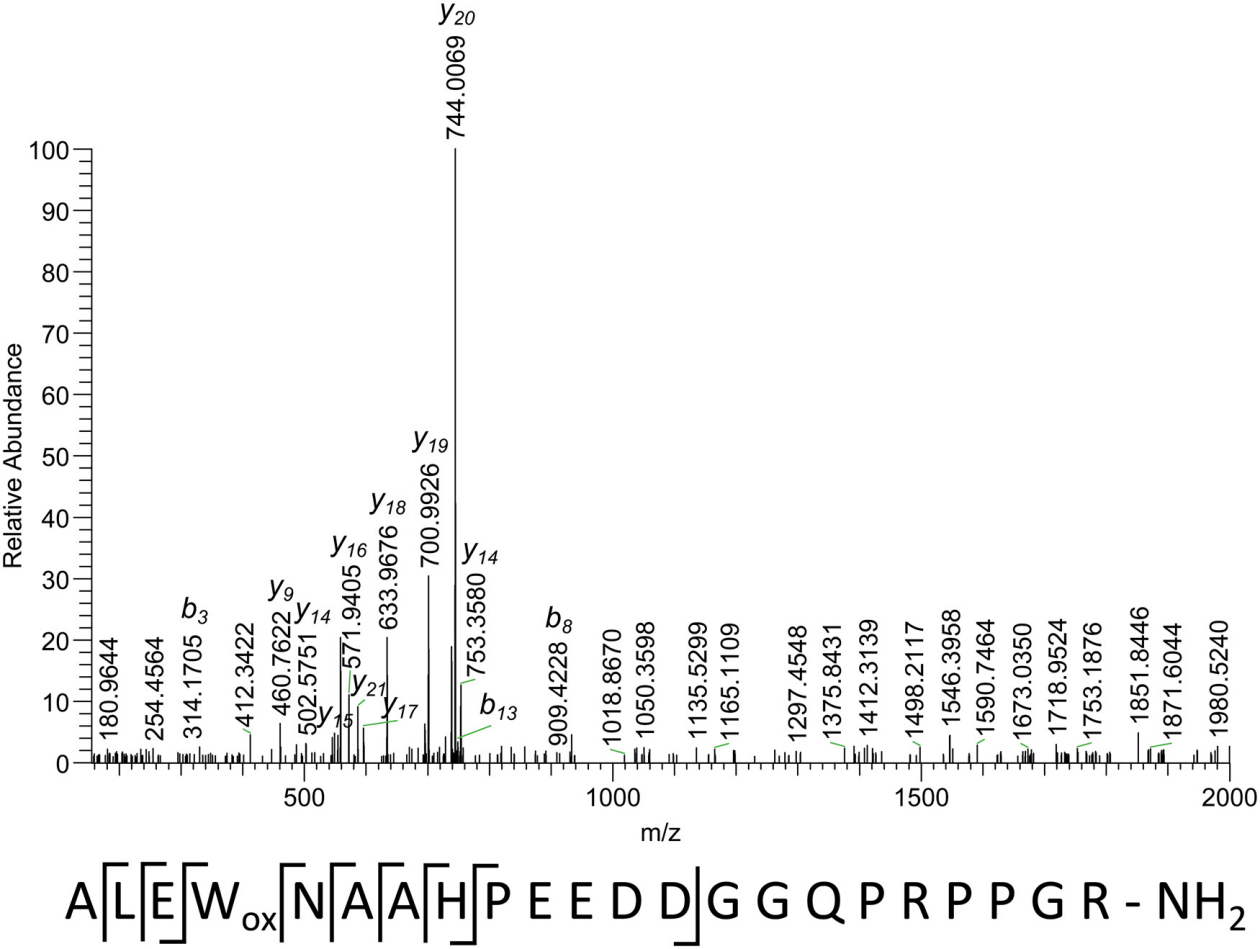
Fragmentation spectrum of a peptide with accurate measured mass of 2414.1245 Da (M), quadruply-charged precursor (m/z 604.2866). The sequence under the spectrum shows observed cleavages.

Most of the observed cleavages in the spectrum in Fig 4 represent fragmentation of the N-terminal part of the peptide. The interesting feature of this spectrum is an unusual mass difference between y_18_ and y_19_ (202.0788 Da), which can be attributed either to a VC or an AM pair (5.95 ppm). During database matching none of these sequences was found, however a closely related sequence can be found: ALEWNAAH. The mass difference can be nicely explained as oxidation of tryptophan to hydroxytryptophan (indicated as W_ox_ in the sequence). The difference between theoretical and experimental mass is +0.0046 Da. The resulting three hits are related to the same three proteins, as in the previous case (Figs 2 and 3), while the peptide sequence is ALEWNAAHPEEDDGGQPRPPGR. The difference between the masses of peptides in the database and the measured mass is -0.9825 Da. Since this peptide is related to the same proteins it is reasonable to assume a C-terminal amidation leading to the mass shift. Mass errors for the C-terminal non-sequenced part and the whole peptide are 1.01 ppm and 0.53 ppm, respectively.

**Fig 5:**
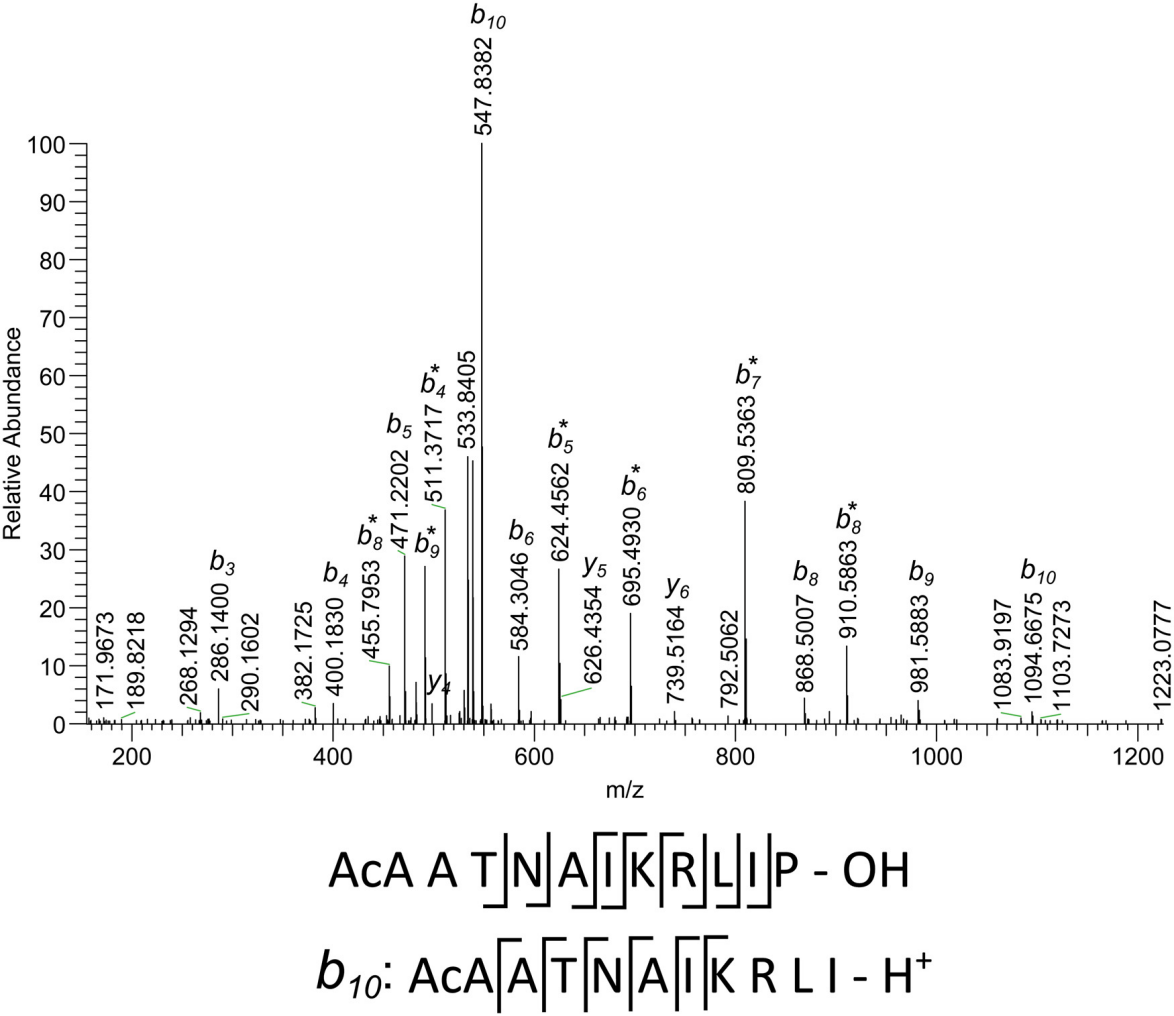
Fragmentation spectrum of a peptide with accurate measured mass of 1209.7315 Da (M), doubly-charged precursor (m/z 605.3694). The sequence under the spectrum shows observed cleavages for complete peptide and for b10-ion. Internal ions formed from b10 are marked with asterisk.

The fragmentation spectrum presented in Fig 5 allows establishing the C-terminal part of the peptide sequence, but the N-terminal part remains concealed—there are 5 possible amino acid compositions for b_3_-ion (286.1398 Da). The striking peculiarity of this spectrum is the high intensity of internal b-ions. Using them it is possible to find the structure for b_10_ (1094.6705 Da) as AcATNAIKRLI-H+. The smallest of the internal ions having a mass of 511.3717 Da can have only one amino acid composition—KRLL, while for the first position in the sequence (113.0501 Da) there are two possible explanations—hydroxyproline (Hyp) or N-acetylated alanine (AcA). The complete peptide sequence can be established as (AcA|Hyp)ATNALKRLLP-OH (both leucine and isoleucine are indicated with L). This sequence can be found in the 10 kDa heat shock protein. The mass error is 0.08 ppm for the complete peptide.

**Fig 6:**
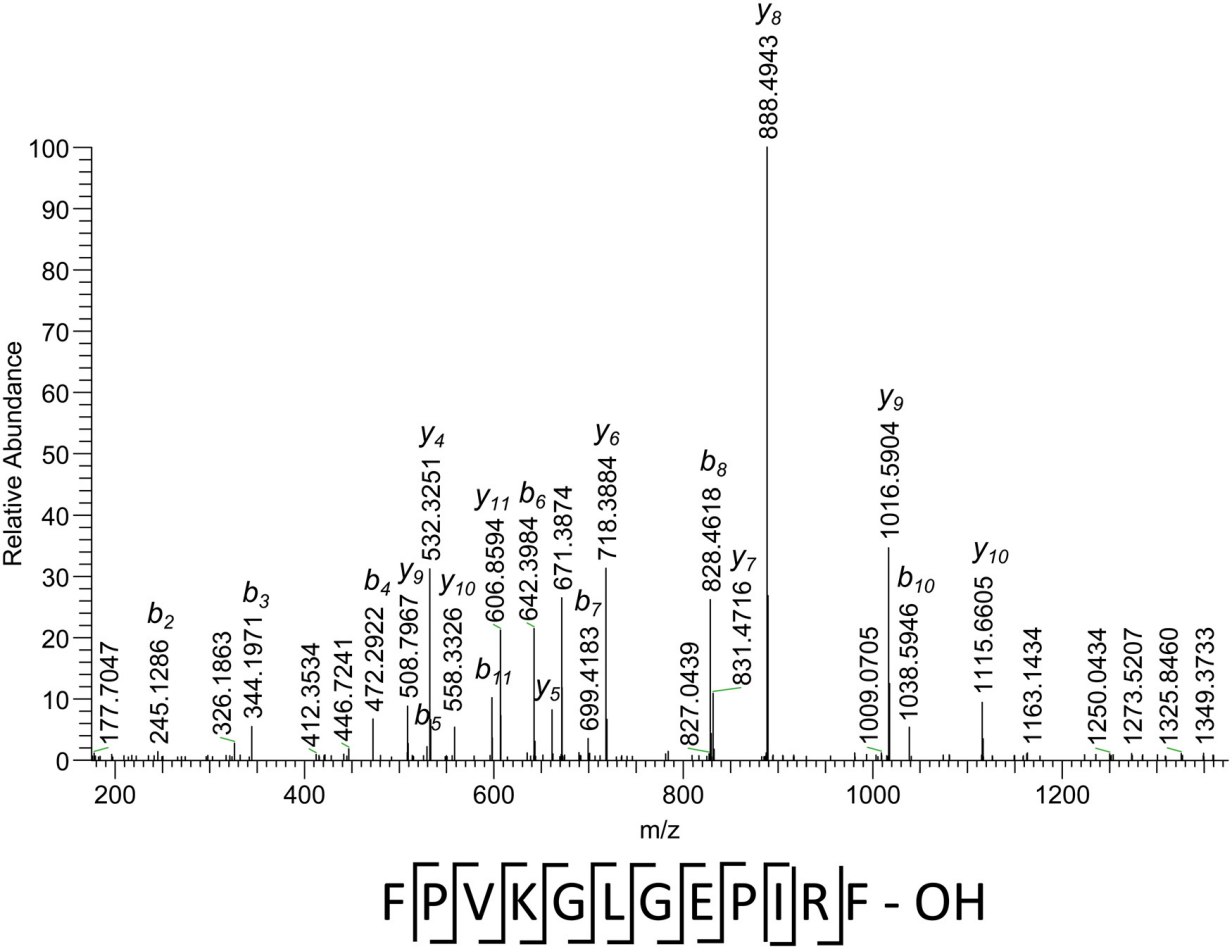
Fragmentation spectrum of a peptide with accurate measured mass of 1359.7779 Da (M), doubly-charged precursor (m/z 680.3926). The sequence under the spectrum shows observed cleavages.

Fig 6 shows the fragmentation spectrum of the peptide from vesicula seminalis, having accurate measured mass of 1359.7779 Da (uncharged). Using the quite prominent and long series of y-ions, supported by complementary b-ions (Fig 6), it is possible to establish the complete sequence of the peptide, excluding L/I identification, since the latter is not possible with the fragmentation method applied. This sequence is not present in any of the known proteins in the database, though the best match (FNVMGLGEPIRF) can be found in three proteins, annotated as Glutathione S-transferase S4. The difference between sequences is two single amino acid modifications at positions 2 and 4, namely N-> P and M-> K. Both substitutions are quite rare in proteins and, therefore, can be of some interest. The mass error for complete peptide is 1.10 ppm.

The complete list of all proteins found in male accessory glands of the bee drone is presented in Table 2 (Additional details can be found in S2 Table). The number of identified peptides and proteins is almost doubled after the *de novo* sequencing step (131 peptides, 50 proteins, 2.6 peptides/protein). Some of the identified peptides contain at least one modification (see S2 Table). All newly added peptides can be found at least in one protein from the database. However, in several cases new sequences can be attributed to more than one protein, but these proteins seem to be homologous and share the same function. *De novo* sequencing complements the database search results not only by adding new proteins, but also by giving additional support for the proteins found by SEQUEST and thus providing more than one peptide evidence per protein.

**Table 2:** Proteins identified in reproduction-related glands of *Apis mellifera* drones by SEQUEST and *de novo* approach.

Uniprot	RefSeq	BeeBase	Description	Function	Peptides (DB/Total)	B	C	V
**A5A5E4**	-	GB46297-PA	Structural cuticle protein (Fragment) OS = Apis mellifera PE = 2 SV = 1	Structural molecule activity	**8/20**	X	X	
**H9KBS5**	NP_001257759	GB52161-PA	PREDICTED: flexible cuticle protein 12 OS = Apis mellifera GN = CPR28 PE = 4 SV = 1	Structural molecule activity	**5/8**	X	X	
H9KGM8	-	GB46297-PA	PREDICTED: endocuticle structural glycoprotein SgAbd-2-like OS = Apis mellifera	Structural molecule activity	17/17		X	
H9K0P8	XP_001120021	GB44777-PA	PREDICTED: hypothetical protein LOC725882 OS = Apis mellifera GN = LOC725882 PE = 4	Molybdopterin biosynthesis protein MoeB domain; Validated	1/1		X	
**H9KU41**	-	GB46312-PA	PREDICTED: endocuticle structural glycoprotein SgAbd-8 OS = Apis mellifera	Structural molecule activity	**8/11**	X	X	
**Q6VQ13**	NP_001010975	GB42422-PA	ADP/ATP translocase OS = Apis mellifera GN = Ant PE = 2 SV = 1	Transporter activity, cytoplasm, membrane, mitochondrial	**5/8**	X		X
H9KHD2	XP_392478	GB42526-PA	PREDICTED: malate dehydrogenase, mitochondrial-like isoform1 OS = Apis mellifera	Carbohydrate metabolism, citric acid cycle	2/2			X
H9KC10	XP_001120194	GB45913-PA	PREDICTED: protein lethal(2)essential for life-like OS = Apis mellifera GN = LOC724488 PE = 3	Heat-shock protein 20 (HSP20)	1/1			X
H9KJ51	-	GB55598-PA	Troponin I OS = Apis mellifera PE = 4 SV = 1	Muscle constriction, part of troponin complex, binds actin	1/1			X
**H9KA48**	-	GB40759-PA	Icarpin precursor OS = Apis mellifera PE = 4 SV = 1	Venom carbohydrate-rich protein, secreted by venom duct, extracelular	**1/2**		X	
H9KMZ3	XP_625049	GB48244-PA	Cyclin-dependent kinase 6-like OS = Apis mellifera PE = 4 SV = 1	Kinase activity, phosphorylation, cell cycle regulation	1/1			X
H9KTW5	XP_624662	GB48904-PA	PREDICTED: glutathione S-transferase-like OS = Apis mellifera GN = LOC552283 PE = 3	Transferase, processing of xenobiotics, detoxification, diverse functions	1/1			X
H9K5H8	XP_392114	GB51551-PA	PREDICTED: myophilin OS = Apis mellifera GN = Chd64 PE = 4 SV = 1	Muscle associated	1/1			X
H9K6R4	XP_001121551	GB46094-PA	PREDICTED: actin, cytoplasmic 1-like, partial OS = Apis mellifera GN = LOC725739 PE = 4	Muscle constriction	1/1			X
**H9K538**	XP_393297	GB42792-PA	PREDICTED: hypothetical protein LOC409805 OS = Apis mellifera GN = Mf PE = 4 SV = 1	Unknown	**2/3**			X
H9KIT9	XP_624806	GB43999-PA	PREDICTED: peroxiredoxin-5, mitochondrial OS = Apis mellifera GN = Prx5 PE = 4 SV = 1	Response to oxidative stress, cell redox homeostasis	2/2		X	X
B0LUE8	NP_001107670	GB55452-PA	Apolipophorin-III-like protein OS = Apis mellifera GN = A4 PE = 2 SV = 1	Lipid transport, extracelular	1/1		X	
H9KMZ2	-	-	PREDICTED: hypothetical protein LOC552453 OS = Apis mellifera GN = LOC552453 PE = 4	Conserved Domain: Protein of unknown function (DUF745); Drosophila proteins family	1/1			X
**H9K1E2**	XP_006559246	GB54446-PA	Arginine kinase OS = Apis mellifera GN = Argk PE = 3 SV = 1	Phosphorylation, arginine metabolism, proline metabolism	**1/4**	X		X
H9KKD2	XP_001121746	GB53748-PA	PREDICTED: hypothetical protein LOC725960 OS = Apis mellifera GN = LOC725960 PE = 4	Unknown	1/1		X	
H9K7H5	XP_001122696	GB42797-PA	PREDICTED: protein takeout-like OS = Apis mellifera GN = LOC726981 PE = 4 SV = 1	Probably: chemoreception; Probably: circadian-controled	1/1		X	
H9KGE6	XP_393789	GB47268-PA	PREDICTED: electron transfer flavoprotein subunit beta-like OS = Apis mellifera	Phosphorylation, electron transport, microtubule associated, lipid particle	1/1		X	
Q5XUU6	NP_001011640	GB48492-PA	Take-out-like carrier protein JHBP-1 OS = Apis mellifera PE = 2 SV = 1	Transfer of Juvenile Hormone PFAM: Pf06585	1/1		X	
H9KR59	XP_006568820	GB55482-PA	PREDICTED: Na(+)/H(+) exchange regulatory cofactor NHE-RF1-like OS = Apis mellifera	PDZ signaling domain: protein-protein interaction, signaling	1/1			X
H9KP88	-	GB54676-PA	Aspartate aminotransferase OS = Apis mellifera GN = Ame.2149 PE = 3 SV = 1	Aminoacid metabolism, alkaloid synthesis	1/1			X
H9K024	XP_006567025	GB52107-PA	Tubulin alpha OS = Apis mellifera GN = LOC408388 PE = 3 SV = 1	Microtubule comp., protein polymeryzation, antimicrobial humoral response	1/1			X
H9K626	XP_393545	GB52073-PA	PREDICTED: probable citrate synthase 1, mitochondrial-like OS = Apis mellifera	Carbohydrate metabolism, citric acid cycle	1/1			X
**H9KNB7**	XP_003251464	GB41306-PA	Actin OS = Apis mellifera GN = LOC410075 PE = 3 SV = 1	Muscle constriction	**1/3**			X
**H9KPL4**	XP_392899	GB54372-PA	PREDICTED: 60 kDa heat shock protein, mitochondrial-like OS = Apis mellifera	Response to stress, protein refolding, transmembrane transport, pH regulation	**1/3**			X
H9K918	XP_006564892	GB52736-PA	Probably: ATP synthase subunit beta OS = Apis mellifera GN = Atp5b PE = 3 SV = 1	Oxidative phosporylation, proton transport, ATP synthesis	1/1		X	
H9KR70	XP_625171	GB53437-PA	PREDICTED: f-box only protein 32-like OS = Apis mellifera PE = 4 SV = 1	Cell cycle regulation, signal transduction	1/1			X
***H9KUC2***	NP_001257753	GB52202-PA	Cuticular protein 12 precursor [Apis mellifera]	Structural molecule activity	***0/1***	X		
***H9K3K6***	XP_623787	GB50875-PA	PREDICTED: apolipoprotein D-like isoform 2 [Apis mellifera]	Lipid transport, lipid binding, pigment binding	***0/1***	X		
***H9KCL3***	-	GB53775-PA	PREDICTED: retinol dehydrogenase 14-like [Apis mellifera]	Oxidoreductase activity	***0/1***	X		
***H9K639***	-	GB47336-PA	PREDICTED: protein disulfide-isomerase [Apis mellifera]	Protein folding, disulfide formation. Location: extracellular, EPR	***0/1***	X		
***H9JYV0***	-	GB53065-PA	PREDICTED: 60S ribosomal protein L7 [Apis mellifera]	Strctural constituent of ribosome	***0/1***	X		
***H9KEY1***	XP_392760	GB55643-PA	PREDICTED: ATP synthase subunit O, mitochondrial [Apis mellifera]	ATP synthesis, pfam00213	***0/3***	X	X	X
***H9KAU3***	XP_624910	GB54343-PA	PREDICTED: 10 kDa heat shock protein, mitochondrial-like [Apis mellifera]	Response to stress, protein folding	***0/4***		X	X
***H9K748 H9KU35***	-	GB51653-PA	PREDICTED: myosin heavy chain, muscle [Apis mellifera] Also: H9KU34, H9KU33, H9KU31	Motor activity, actin binding	***0/5***			X
***H9KUH8 H9KLF7***	XP_006559096	GB51800-PA	PREDICTED: hypothetical protein LOC409090 [Apis mellifera]	ATPase inhibitor domain, metabolic process regulation, pfam04568, pfam13873	***0/1***			X
***H9KHG2***	XP_001121882	GB52644-PA	PREDICTED: ATP synthase-coupling factor 6, mitochondrial [Apis mellifera]	Mitochondrial proton-transporting ATP synthase complex	***0/1***			X
***H9KN88***	-	GB40460-PA	PREDICTED: probable enoyl-CoA hydratase, mitochondrial [Apis mellifera]	Fatty acid beta-oxidation, fatty acid metabolism	***0/1***			X
***H9K4S1***	XP_393900	GB48255-PA	PREDICTED: isochorismatase domain-containing protein 1-like [Apis mellifera]	Hydrolase activity, metabolic process	***0/1***			X
***H6CSZ2 H9KBB1***	-	GB49545-PA	Glutathione S-transferase S4 [Apis mellifera] Also: B6VCW8	Transferase activity	***0/2***			X
***H9K8G6***	XP_006558384	GB40240-PA	PREDICTED: myosin regulatory light chain 2 [Apis mellifera]	Ca-binding, superoxide metabolism, oxidation-reduction process	***0/1***			X
***H9KPS8***	XP_624401	GB50595-PA	PREDICTED: aldo-keto reductase, NADP+ dependent [Apis mellifera]	Oxidoreductase activity	***0/1***			X
***H9K0L2***	XP_393300	GB48536-PA	PREDICTED: t-complex protein 1 subunit beta-like isoform 1 [Apis mellifera]	Protein folding, Chaperone	***0/1***			X
***H9KR11***	XP_006563323	GB40735-PA	PREDICTED: fructose-bisphosphate aldolase-like [Apis mellifera]	Glycolysis	***0/1***			X
***H9K9A4***	XP_001120471	GB55232-PA	PREDICTED: 3-hydroxyacyl-CoA dehydrogenase type-2-like [Apis mellifera]	Oxidoreductase activity, dehydrogenase-reductase	***0/1***			X

Uniprot accession number, functional description, number of peptides supporting the protein found during database step (DB) and database + *de novo* step (Total) and the origin are provided for each protein. Proteins received additional support during *de novo* step are marked with bold font, proteins found only during *de novo* step are marked with bold-italic font. B—bulbus gland, C—cornua gland, V—vesicula seminalis.

The distribution of proteins found in each gland is as follows: bulbus−12 (6 unique) proteins, cornua−15 (7 unique) proteins, vesicula seminalis−32 (28 unique) proteins. It should be mentioned, that most of the proteins are unique for a specific gland type, only one of them (H9KEY1/ATP synthase subunit O) was found in all three glands, 7 proteins were found in the two types of glands which contribute to the mating sign. The major part of identified proteins originates from vesicula seminalis, the only secretion which contributes to the seminal fluid.

The main functional classes of found proteins are the same as reported earlier [21,30,34], but the direct comparison of protein lists shows only partial overlap (Table 3). Collins et al. [21] investigated the protein composition of semen and seminal vesicles, and 8 proteins from the current investigation were found in their work as well. The tissue of origin matches our observation in all cases, except for ATP synthase (H9K918) identified only in the cornua gland. Baer et al. [30] focused on seminal-fluid protein composition, and there are only two proteins in common with our findings. Arginine kinase (H9K1E2) was found in the bulbus gland and the seminal vesicles; the second one is citrate synthase 1 which was identified in seminal vesicles. Chan et al. [34] published a protein atlas of the honey bee that included proteomic analysis of complete bee drone testis and mucus gland. The contents of mucus gland were investigated by us as well; however no proteins could be identified. There is little overlap between their observations and earlier reports [21,30] (Table 3). Twenty proteins identified in the current investigation were also found in the analysis of Chan et al.[34]. The vast majority of these proteins were present in both organs (testis and mucus gland), only three proteins (H9K6R4, H9KDD2 and Q5XUU6) are located in testis only. Since testis was analyzed as one sample, it is not possible to compare gland localization of identified proteins. According to our observations, most of the common proteins are present in vesicula seminalis. The only protein identified in all three glands (ATP synthase, H9KEY1) was reported by Chan et al. as well [34]. Although the overlap with the Bee protein atlas [34] is quite sound, a significant part of the proteins found in the current investigation was not reported earlier. The functional variety of overlapping proteins is quite broad and includes protein folding, carbohydrate and lipid metabolism, phosphorylation/phosphate metabolism, oxidoreductase activity, and structural activity.

**Table 3:** Direct comparison of identified proteins with those reported earlier [21,30,34].

Accession	BeeBase	Description	Function	Origin (1)	Origin (2)	Origin (3)	B	C	V
B0LUE8	GB55452-PA	Apolipophorin-III-like protein OS = Apis mellifera GN = A4 PE = 2 SV = 1	Lipid transport, extracelular			T+M		X	
H9K024	GB52107-PA	Tubulin alpha OS = Apis mellifera GN = LOC408388 PE = 3 SV = 1	Microtubule comp., protein polymeryzation, humoral response		Se+VS				X
H9K0L2	GB48536-PA	PREDICTED: t-complex protein 1 subunit beta-like isoform 1 [Apis mellifera]	Protein folding, Chaperone			T+M			X
H9K1E2	GB54446-PA	Arginine kinase OS = Apis mellifera GN = Argk PE = 3 SV = 1	Phosphorylation, arginine metabolism, proline metabolism	SF	Se+VS		X		X
H9K4S1	GB48255-PA	PREDICTED: isochorismatase domain-containing protein 1-like [Apis mellifera]	Hydrolase activity, metabolic process			T+M			X
H9K5H8	GB51551-PA	PREDICTED: myophilin OS = Apis mellifera GN = Chd64 PE = 4 SV = 1	Muscle associated			T+M			X
H9K626	GB52073-PA	PREDICTED: probable citrate synthase 1, mitochondrial-like OS = Apis mellifera	Carbohydrate metabolism, citric acid cycle	SF		T+M			X
H9K6R4	GB46094-PA	PREDICTED: actin, cytoplasmic 1-like, partial OS = Apis mellifera GN = LOC725739 PE = 4	Muscle constriction			T			X
H9K8G6	GB40240-PA	PREDICTED: myosin regulatory light chain 2 [Apis mellifera]	Ca-binding, superoxide metabolism, oxidation-reduction process		VS				X
H9K918	GB52736-PA	Probably: ATP synthase subunit beta OS = Apis mellifera GN = Atp5b PE = 3 SV = 1	Oxidative phosporylation, proton transport, ATP synthesis		VS			X	
H9K9A4	GB55232-PA	PREDICTED: 3-hydroxyacyl-CoA dehydrogenase type-2-like [Apis mellifera]	Oxidoreductase activity, dehydrogenase-reductase			T+M			X
H9KAU3	GB54343-PA	PREDICTED: 10 kDa heat shock protein, mitochondrial-like [Apis mellifera]	Response to stress, protein folding			T+M		X	X
H9KC10	GB45913-PA	PREDICTED: protein lethal(2)essential for life-like OS = Apis mellifera GN = LOC724488 PE = 3	Heat-shock protein 20 (HSP20)			T+M			X
H9KEY1	GB55643-PA	PREDICTED: ATP synthase subunit O, mitochondrial [Apis mellifera]	ATP synthesis, pfam00213			T+M	X	X	X
H9KGE6	GB47268-PA	PREDICTED: electron transfer flavoprotein subunit beta-like OS = Apis mellifera	Phosphorylation, electron transport, microtubule associated, lipid particle			T+M		X	
H9KHD2	GB42526-PA	PREDICTED: malate dehydrogenase, mitochondrial-like isoform1 OS = Apis mellifera	Carbohydrate metabolism, citric acid cycle			T+M			X
H9KHG2	GB52644-PA	PREDICTED: ATP synthase-coupling factor 6, mitochondrial [Apis mellifera]	Mitochondrial proton-transporting ATP synthase complex			T+M			X
H9KIT9	GB43999-PA	PREDICTED: peroxiredoxin-5, mitochondrial OS = Apis mellifera GN = Prx5 PE = 4 SV = 1	Response to oxidative stress, cell redox homeostasis			T+M		X	X
H9KKD2	GB53748-PA	PREDICTED: hypothetical protein LOC725960 OS = Apis mellifera GN = LOC725960 PE = 4	Unknown			T		X	
H9KNB7	GB41306-PA	Actin OS = Apis mellifera GN = LOC410075 PE = 3 SV = 1	Muscle constriction		VS				X
H9KPL4	GB54372-PA	PREDICTED: 60 kDa heat shock protein, mitochondrial-like OS = Apis mellifera	Response to stress, protein refolding, transmembrane transport		VS	T+M			X
H9KPS8	GB50595-PA	PREDICTED: aldo-keto reductase, NADP+ dependent [Apis mellifera]	Oxidoreductase activity		VS	T+M			X
H9KR11	GB40735-PA	PREDICTED: fructose-bisphosphate aldolase-like [Apis mellifera]	Glycolysis		VS				X
H9KTW5	GB48904-PA	PREDICTED: glutathione S-transferase-like OS = Apis mellifera GN = LOC552283 PE = 3	Transferase, processing of xenobiotics, detoxification, diverse functions			T+M			X
Q5XUU6	GB48492-PA	Take-out-like carrier protein JHBP-1 OS = Apis mellifera PE = 2 SV = 1	Transfer of Juvenile Hormone PFAM: Pf06585			T		X	
Q6VQ13	GB42422-PA	ADP/ATP translocase OS = Apis mellifera GN = Ant PE = 2 SV = 1	Transporter activity, cytoplasm, membrane, mitochondrial			T+M	X		X

(1) – [30]; (2) – [21]; (3) – [34]; SF – seminal fluid; Se – semen; T – testis; M – mucus gland;B – bulbus gland; C – cornua gland; VS – vesicula seminalis.

Five proteins found in bulbus and cornua glands (A5A5E4, H9KBS5, H9KGM8, H9KU41, H9KUC2), both by *de novo* and database methods, are attributed as cuticular proteins. Both glands are of ectodermal origin and bulbus glands produce chitinous plates [18]. The remaining proteins can be separated to the same functional groups as reported by Baer et al [29,30] and Collins et al [21]. The defense group includes proteins involved in degradation of potentially harmful substances, e.g. glutathione S-transferases, peroxiredoxin, and several oxidoreductases. One of the glutathione S-transferases belongs to the sigma class. It is known that members of this class have high activity for lipid peroxidation products and were described in metabolically active tissues (e.g. flight muscles) in flies, in bee venom [35] and also in queen spermatheca and drone semen [12]. The proteins of this group are found mostly in seminal vesicles, the only gland contributing to the production of ejaculate.

It was shown that sugars and phospholipids are the primary sources of energy for sperm [36,37]. Therefore, the presence of proteins involved in degradation of side-products looks quite reasonable in vesicula seminalis. Proteins involved in metabolism of carbohydrates and lipids were found as well, e.g., malate dehydrogenase, apolipoproteins, citrate synthase, enoyl-CoA hydratase and others. The seminal vesicles contain proteins associated with carbohydrate metabolism, while bulbus and cornua glands contain apolipoproteins (one type each). Moors and Billen [26] reported that lipids form the major part of cornual secretion. The identification of apolipoprotein-III-like protein and electron transfer flavoprotein subunit beta-like supports this observation. The adjacent group of identified proteins involved in energy generation includes arginine kinase, aspartate aminotransferase, fructose-bisphosphate aldolase, citrate synthase, and ATP-related proteins. The largest number of them was found in seminal vesicles, indicating high energy demand of the sperm, however the smaller number of proteins were found in bulbus and cornua gland as well. The major part of protein-processing proteins, e.g., heat shock proteins and disulphate isomerase were identified in seminal vesicles. They might be involved in conditioning of the seminal fluid environment. Several representatives of this class were also found in bulbus and cornua glands. A relatively large group of proteins found almost exclusively in seminal vesicles belong to the structural/motor group, like myosin, actin, tubulin, troponin, the representatives of this class were also reported by Collins and Baer [21,30]. The group of signaling proteins consists of circadian-controlled protein (H9K7H5) and juvenile hormone transporter which were both found in the cornua gland. This type of proteins might mediate female controlling functions of sperm. The function of some identified proteins remains uncertain and might be the target of further investigations.

The possibility of all proteins being secreted was analyzed by TargetP [38]. Eleven proteins were identified as secreted ones and two contained a mitochondrion transport signal. The localization of the remaining proteins cannot be predicted. Thus, only about 20% of the proteins are predicted to be secreted. A similar ratio was also found for human and bee seminal proteins in earlier studies [30]. Prediction results are provided in Table 4.

**Table 4:** Localization prediction of identified proteins by TargetP [38].

Accession	BeeBase	Description	Localization	B	C	V
A5A5E4	GB46297-PA	Structural cuticle protein (Fragment) OS = Apis mellifera PE = 2 SV = 1	Secretory	X	X	
H9KBS5	GB52161-PA	PREDICTED: flexible cuticle protein 12 OS = Apis mellifera GN = CPR28 PE = 4 SV = 1	Secretory	X	X	
H9KGM8	GB46297-PA	PREDICTED: endocuticle structural glycoprotein SgAbd-2-like OS = Apis mellifera	Secretory		X	
H9KA48	GB40759-PA	Icarpin precursor OS = Apis mellifera PE = 4 SV = 1	Secretory		X	
H9KIT9	GB43999-PA	PREDICTED: peroxiredoxin-5, mitochondrial OS = Apis mellifera GN = Prx5 PE = 4 SV = 1	Mitochondrion		X	X
B0LUE8	GB55452-PA	Apolipophorin-III-like protein OS = Apis mellifera GN = A4 PE = 2 SV = 1	Secretory		X	
H9KKD2	GB53748-PA	PREDICTED: hypothetical protein LOC725960 OS = Apis mellifera GN = LOC725960 PE = 4	Secretory		X	
H9K7H5	GB42797-PA	PREDICTED: protein takeout-like OS = Apis mellifera GN = LOC726981 PE = 4 SV = 1	Secretory		X	
Q5XUU6	GB48492-PA	Take-out-like carrier protein JHBP-1 OS = Apis mellifera PE = 2 SV = 1	Secretory		X	
H9KPL4	GB54372-PA	PREDICTED: 60 kDa heat shock protein, mitochondrial-like OS = Apis mellifera	Mitochondrion			X
H9KUC2	GB52202-PA	Cuticular protein 12 precursor [Apis mellifera]	Secretory	X		
H9K3K6	GB50875-PA	PREDICTED: apolipoprotein D-like isoform 2 [Apis mellifera]	Secretory	X		
H9K639	GB47336-PA	PREDICTED: protein disulfide-isomerase [Apis mellifera]	Secretory	X		

B—bulbus gland, C—cornua gland, V—vesicula seminalis.

The list of potentially secreted proteins includes all cuticle proteins, icarpin, apolipoproteins which are associated with lipid metabolism, both proteins with signaling functions, and disulfide-isomerase which belongs to the protein processing group.

It should be mentioned specifically that the prediction of secretory proteins is based solely on the presence or absence of some specific amino acid motifs in the protein sequence, which target it to the specific pathway. However, several other factors could influence the behavior of the protein, not mentioning the potential inaccuracy of the prediction.

## Conclusions

The content of four different reproduction-related glands of the honeybee drone (bulbus, cornua, mucus and seminal vesicles) were analyzed separately, while proteins were found only in three of them, excluding the mucus gland. The combination of database search and *de novo* sequencing allowed the identification of a total of 50 proteins. The *de novo* sequencing approach significantly increased the amount of peptides and proteins identified in the samples and allowed establishing peptide modifications not included during database search. The largest number of proteins was found in seminal vesicles, the only gland that contributes to semen proteins. There are not many proteins in common between the various gland types. Most of them were found in one specific gland only. Comparing to earlier investigations on seminal fluid of bee drone and the content of some reproductive glands [21,30] it should be mentioned that most of the proteins found in this study weren’t reported earlier. All proteins can be subdivided in the following functional groups: defense group that includes proteins involved in degradation of potentially harmful substances, proteins involved in metabolism of carbohydrates and lipids, proteins involved in energy generation, proteins with protein processing and protein conditioning functions, structural/motor proteins, signaling proteins, and several proteins with unknown function. Defense, carbohydrate metabolism, significant part of energy production related, motor and protein conditioning proteins were found to be located in seminal vesicles, indicating the necessity to provide male gametes with energy and protection. Cuticle proteins are unique for bulbus and cornua glands and might be related to the glands production of chitinous plates. Moreover, both cornua and bulbus glands contain lipid metabolism proteins, the representatives of this functional class were not found in other glands. The unique feature of cornua glands is the presence two signaling proteins. Using TargetP [38], 11 proteins were predicted to be secreted and 2 proteins to be located in mitochondria. The secreted group contains proteins involved in lipid metabolism, protein processing ones and both signaling proteins.

## Supporting Information

S1 Table Detailed list of proteins identified only by database search (SEQUEST) in reproduction related glands of the bee (*A*. *mellifera*) drone. (DOCX)

S2 Table Detailed list of proteins identified by combination of database search (SEQUEST) and *de novo* sequencing in reproduction-related glands of the bee (*A*. *mellifera*) drone. (DOCX)

S1 Text Description of the home-built supporting software. (DOCX)

S2 Text Comparison of SEQUEST and *de novo* results for the same peptide. (DOCX)

S3 Text Examples of *de novo* sequencing. (DOCX)
