# Supplementary material for: Protein and Peptide Composition of Male Accessory Glands of Apis mellifera Drones Investigated by Mass Spectrometry
Source: PLoS One. 2015 May 8;10(5):e0125068. doi: 10.1371/journal.pone.0125068 (PMC4425483; doi:10.1371/journal.pone.0125068)
Supplement: S1 Table — (DOCX) [file pone.0125068.s001.docx]

**Supporting Information Table S3. Detailed list of proteins identified only by database search (SEQUEST) in reproduction-related glands of the bee (*A. mellifera*) drone.**

| **Accession** | **Description** | **Sequences** | **Modifications** | **MH+ [Da]** | **PEP** |
| --- | --- | --- | --- | --- | --- |
| A5A5E4 | Structural cuticle protein (Fragment) | APQRPSGGADKDAVITSQQL |  | 2039.05069 | 0.000696815 |
|  |  | VADENGFQVQGSHIPTAPPIPPEIQR |  | 2797.42619 | 1.37531E-06 |
|  |  | AAHPEEDDGGQPRPPGRG |  | 1842.84793 | 0.008416717 |
|  |  | APQRPSGGADKDAVITSQQ |  | 1925.96745 | 0.04085944 |
|  |  | VADENGFQVQGSHIPTAPPIPPEIQRA |  | 2868.46536 | 0.00214772 |
|  |  | SGGADKDAVITSQQL |  | 1489.74873 | 0.113153 |
|  |  | APQRPSGGADKDAVITSQ |  | 1797.90888 | 0.01163706 |
|  |  | SGGADKDAVITSQ |  | 1248.60640 | 0.2068612 |
| H9KBS5 | PREDICTED: flexible cuticle protein 12 | IIKQEEQNNIGVGGY |  | 1661.84919 | 2.92146E-06 |
|  |  | HFSYEQSDGQKREET |  | 1840.80982 | 0.00162104 |
|  |  | RVDYTADKDGFHPTINL |  | 1961.97230 | 2.12586E-06 |
|  |  | HFSYEQSDGQKREETAE |  | 2040.88996 | 6.02924E-06 |
|  |  | IIKQEEQNNIGVGGYHF |  | 1945.97685 | 0.00089221 |
| H9KGM8 | PREDICTED: endocuticle structural glycoprotein SgAbd-2-like | RALDWIVANPEKNRL |  | 1794.99781 | 4.0804E-06 |
|  |  | NQDISPDGTFHSK |  | 1445.66569 | 1.50212E-05 |
|  |  | APQRPSGGADKDAVITSQQL |  | 2039.05069 | 0.000696815 |
|  |  | VADENGFQVQGSHIPTAPPIPPEIQR |  | 2797.42619 | 1.37531E-06 |
|  |  | ALDWIVANPEKNRL |  | 1638.89639 | 0.01064163 |
|  |  | ADQPIAIIRQ |  | 1124.64207 | 0.005068009 |
|  |  | FEEEGVLKN |  | 1064.52602 | 0.1136837 |
|  |  | ALDWIVANPEKN |  | 1369.71180 | 0.03313574 |
|  |  | ADQPIAIIR |  | 996.58373 | 0.001606068 |
|  |  | ALDWIVANPEKNR |  | 1525.81202 | 0.01481048 |
|  |  | APQRPSGGADKDAVITSQQ |  | 1925.96745 | 0.04085944 |
|  |  | VADENGFQVQGSHIPTAPPIPPEIQRA |  | 2868.46536 | 0.00214772 |
|  |  | SGGADKDAVITSQQL |  | 1489.74873 | 0.113153 |
|  |  | NQDISPDGTFHSKWESANG |  | 2089.92123 | 0.002030485 |
|  |  | APQRPSGGADKDAVITSQ |  | 1797.90888 | 0.01163706 |
|  |  | SGGADKDAVITSQ |  | 1248.60640 | 0.2068612 |
|  |  | LDWIVA |  | 716.39773 | 0.2450954 |
| H9K0P8 | PREDICTED: hypothetical protein LOC725882 | FEILGAHVPSEVVNNL |  | 1737.91744 | 0.01099127 |
| H9KU41 | PREDICTED: endocuticle structural glycoprotein SgAbd-8 | ALDWIAAHPSKEDQNQV |  | 1921.94000 | 0.000902929 |
|  |  | LDWIAAHPSKEDQNQV |  | 1850.90257 | 0.04484197 |
|  |  | APAEDVIPIVAQ |  | 1222.66783 | 0.08522508 |
|  |  | DWIAAHPSKEDQNQV |  | 1737.81826 | 0.002361879 |
|  |  | LDWIAAHPSKEDQNQ |  | 1751.83442 | 0.0252533 |
|  |  | WIAAHPSKEDQNQV |  | 1622.79168 | 0.1074335 |
|  |  | APAEDVIPIVA |  | 1094.60916 | 0.06286725 |
|  |  | AEDVIPIVAQ |  | 1054.57711 | 0.0342243 |
| Q6VQ13 | ADP/ATP translocase | ISKTTVAPIERVKL |  | 1554.95727 | 0.00945212 |
|  |  | GAFSNILRGTGGAL |  | 1333.72221 | 0.0182609 |
|  |  | SKTTVAPIERVKL |  | 1441.87439 | 0.04603312 |
|  |  | GAFSNILRG |  | 934.51071 | 0.1342027 |
|  |  | GGVDKNTQFL |  | 1078.55260 | 0.1343632 |
| H9KHD2 | PREDICTED: malate dehydrogenase, mitochondrial-like isoform 1 | IAIISNPVNSTVPIASEVLK |  | 2065.19174 | 0.009000827 |
|  |  | AKVAILGASGGIGQPLSL |  | 1651.97426 | 0.01776684 |
| H9KC10 | PREDICTED: protein lethal(2)essential for life-like | IEQTGKPALKENTEEKKEEKKE |  | 2586.36134 | 0.00124537 |
| H9KJ51 | Troponin I | LEEEDKEPKKSEKAEWQTKK |  | 2460.26118 | 0.00178813 |
| H9KA48 | Icarpin precursor | FDNEIPKNQGDVLTA |  | 1660.81641 | 0.1684543 |
| H9KMZ3 | Cyclin-dependent kinase 6-like | VPTGDTIFGKIL |  | 1260.71906 | 0.08263831 |
| H9KTW5 | PREDICTED: glutathione S-transferase-like | HYEENEEIKAAKRK |  | 1744.89768 | 0.02660481 |
| H9K5H8 | PREDICTED: myophilin | SNKGANQSGINFGNTR |  | 1664.81052 | 0.03432356 |
| H9K6R4 | PREDICTED: actin, cytoplasmic 1-like, partial | YDESGPSIVH |  | 1103.50060 | 0.09105903 |
| H9K538 | PREDICTED: hypothetical protein LOC409805 | ALKGTDDIRAPEHTHRPRS |  | 2157.12710 | 0.01440642 |
|  |  | GTDDIRAPEHTHRPRS |  | 1844.91076 | 0.06570255 |
| H9KIT9 | PREDICTED: peroxiredoxin-5, mitochondrial | VTVGEKIPTIDL |  | 1284.74096 | 0.00029498 |
|  |  | VGEKIPTIDL |  | 1084.62504 | 0.08487076 |
| B0LUE8 | Apolipophorin-III-like protein | NNQLQTAATQKS |  | 1303.66038 | 0.000224275 |
| H9KMZ2 | PREDICTED: hypothetical protein LOC552453 | ARASAAASEAKANASRN |  | 1645.83599 | 0.03017332 |
| H9K1E2 | Arginine kinase | GTRGEHTEAEGGIYDISNKR |  | 2190.05366 | 0.005887534 |
| H9KKD2 | PREDICTED: hypothetical protein LOC725960 | KAEKPQEEQQEKKAEEKPDRVT |  | 2625.34849 | 0.000704692 |
| H9K7H5 | PREDICTED: protein takeout-like | TDISNSILKQF |  | 1265.67436 | 0.000100702 |
| H9KGE6 | PREDICTED: electron transfer flavoprotein subunit beta-like | IAKLKESGHI |  | 1095.65291 | 0.1426 |
| Q5XUU6 | Take-out-like carrier protein JHBP-1 | AGGLKSFKIL |  | 1033.64115 | 0.000143763 |
| H9KR59 | PREDICTED: Na(+)/H(+) exchange regulatory cofactor NHE-RF1-like | DKFDIVQKL |  | 1105.62541 | 0.0721139 |
| H9KP88 | Aspartate aminotransferase | TPELKPWVL |  | 1082.62512 | 0.1391333 |
| H9K024 | Tubulin alpha | FSETGAGKHVPRA |  | 1356.70164 | 0.06463558 |
| H9K626 | PREDICTED: probable citrate synthase 1, mitochondrial-like | AAGMNGLAGPLHG |  | 1165.57785 | 0.02117522 |
| H9KNB7 | Actin | YDESGPGIVH |  | 1073.49031 | 0.07256364 |
| H9KPL4 | PREDICTED: 60 kDa heat shock protein, mitochondrial-like | KISKGANPVEIRRG |  | 1524.89786 | 0.04644878 |
| H9K918 | Probably: ATP synthase subunit beta | VVAKAESLAKQ |  | 1143.67363 | 0.1784996 |
| H9KR70 | PREDICTED: f-box only protein 32-like | REVLLR |  | 785.49942 | 0.1391333 |

Each protein is represented by accession number, description and a number of peptides supporting it. Molecular mass, modification and posterior error probability calculated by target-decoy approach are provided for each peptide.
