# Supplementary material for: Protein and Peptide Composition of Male Accessory Glands of Apis mellifera Drones Investigated by Mass Spectrometry
Source: PLoS One. 2015 May 8;10(5):e0125068. doi: 10.1371/journal.pone.0125068 (PMC4425483; doi:10.1371/journal.pone.0125068)
Supplement: S2 Table — (DOCX) [file pone.0125068.s002.docx]

**Supporting Information Table S5. Detailed list of proteins identified by combination of database search (SEQUEST) and *de novo* sequencing in reproduction-related glands of bee (*A. mellifera*) drone.**

| **Accession** | **Description** | **Sequences** | **Modifications** | **MH+ [Da]** |
| --- | --- | --- | --- | --- |
| A5A5E4 | Structural cuticle protein (Fragment) | APQRPSGGADKDAVITSQQL |  | 2039.05069 |
|  |  | VADENGFQVQGSHIPTAPPIPPEIQR |  | 2797.42619 |
|  |  | AAHPEEDDGGQPRPPGRG |  | 1842.84793 |
|  |  | APQRPSGGADKDAVITSQQ |  | 1925.96745 |
|  |  | VADENGFQVQGSHIPTAPPIPPEIQRA |  | 2868.46536 |
|  |  | SGGADKDAVITSQQL |  | 1489.74873 |
|  |  | APQRPSGGADKDAVITSQ |  | 1797.90888 |
|  |  | SGGADKDAVITSQ |  | 1248.60640 |
|  |  | HPEEDDGGQPRPPGR | C-amide | 1642.76740 |
|  |  | EEDDGGQPRPPGR | C-amide | 1408.65680 |
|  |  | AAHPEEDDGGQPRPPGR | C-amide | 1784.84050 |
|  |  | SGGADKDAVITSQQ |  | 1376.66530 |
|  |  | pERPSGGADKDAVITSQQ | pyroGlu | 1740.85070 |
|  |  | pERPSGGADKDAVITSQQL | pyroGlu | 1853.93350 |
|  |  | LEWNAAHPEEDDGGQPRPPGR | C-amide | 2327.09130 |
|  |  | ALEWNAAHPEEDDGGQPRPPGR | C-amide | 2398.12770 |
|  |  | NAAHPEEDDGGQPRPPGR | C-amide | 1898.88570 |
|  |  | WNAAHPEEDDGGQPRPPGR | C-amide | 2084.97210 |
|  |  | ALEWNAAHPEEDDGGQPRPPGR | Trp-OH, C-amide | 2414.12450 |
|  |  | ALEWNAAHPEEDDGGQPRPPGR | C-amide | 2398.12970 |
| H9KBS5 | PREDICTED: flexible cuticle protein 12 | IIKQEEQNNIGVGGY |  | 1661.84919 |
|  |  | HFSYEQSDGQKREET |  | 1840.80982 |
|  |  | RVDYTADKDGFHPTINL |  | 1961.97230 |
|  |  | HFSYEQSDGQKREETAE |  | 2040.88996 |
|  |  | IIKQEEQNNIGVGGYHF |  | 1945.97685 |
|  |  | pEVNPNEITIIKQEEQNN | pyroGlu | 1993.98090 |
|  |  | pEVNPNEITIIK | pyroGlu | 1251.69430 |
|  |  | SFTSPDGHTYR |  | 1267.57040 |
| H9KGM8 | PREDICTED: endocuticle structural glycoprotein SgAbd-2-like | RALDWIVANPEKNRL |  | 1794.99781 |
|  |  | NQDISPDGTFHSK |  | 1445.66569 |
|  |  | APQRPSGGADKDAVITSQQL |  | 2039.05069 |
|  |  | VADENGFQVQGSHIPTAPPIPPEIQR |  | 2797.42619 |
|  |  | ALDWIVANPEKNRL |  | 1638.89639 |
|  |  | ADQPIAIIRQ |  | 1124.64207 |
|  |  | FEEEGVLKN |  | 1064.52602 |
|  |  | ALDWIVANPEKN |  | 1369.71180 |
|  |  | ADQPIAIIR |  | 996.58373 |
|  |  | ALDWIVANPEKNR |  | 1525.81202 |
|  |  | APQRPSGGADKDAVITSQQ |  | 1925.96745 |
|  |  | VADENGFQVQGSHIPTAPPIPPEIQRA |  | 2868.46536 |
|  |  | SGGADKDAVITSQQL |  | 1489.74873 |
|  |  | NQDISPDGTFHSKWESANG |  | 2089.92123 |
|  |  | APQRPSGGADKDAVITSQ |  | 1797.90888 |
|  |  | SGGADKDAVITSQ |  | 1248.60640 |
|  |  | LDWIVA |  | 716.39773 |
| H9K0P8 | PREDICTED: hypothetical protein LOC725882 | FEILGAHVPSEVVNNL |  | 1737.91744 |
| H9KU41 | PREDICTED: endocuticle structural glycoprotein SgAbd-8 | ALDWIAAHPSKEDQNQV |  | 1921.94000 |
|  |  | LDWIAAHPSKEDQNQV |  | 1850.90257 |
|  |  | APAEDVIPIVAQ |  | 1222.66783 |
|  |  | DWIAAHPSKEDQNQV |  | 1737.81826 |
|  |  | LDWIAAHPSKEDQNQ |  | 1751.83442 |
|  |  | WIAAHPSKEDQNQV |  | 1622.79168 |
|  |  | APAEDVIPIVA |  | 1094.60916 |
|  |  | AEDVIPIVAQ |  | 1054.57711 |
|  |  | IAAHPSKEDQNQV |  | 1436.71280 |
|  |  | AEDVIPIVAQ |  | 1054.57810 |
|  |  | AEDVIPIVA |  | 926.51960 |
| Q6VQ13 | ADP/ATP translocase | ISKTTVAPIERVKL |  | 1554.95727 |
|  |  | GAFSNILRGTGGAL |  | 1333.72221 |
|  |  | SKTTVAPIERVKL |  | 1441.87439 |
|  |  | GAFSNILRG |  | 934.51071 |
|  |  | GGVDKNTQFL |  | 1078.55260 |
|  |  | APIERVKL |  | 925.58310 |
|  |  | IPKEQGFLS |  | 1018.55690 |
|  |  | AKDFLAGGVA |  | 948.51430 |
| H9KHD2 | PREDICTED: malate dehydrogenase, mitochondrial-like isoform 1 | IAIISNPVNSTVPIASEVLK |  | 2065.19174 |
|  |  | AKVAILGASGGIGQPLSL |  | 1651.97426 |
| H9KC10 | PREDICTED: protein lethal(2)essential for life-like | IEQTGKPALKENTEEKKEEKKE |  | 2586.36134 |
| H9KJ51 | Troponin I | LEEEDKEPKKSEKAEWQTKK |  | 2460.26118 |
| H9KA48 | Icarpin precursor | FDNEIPKNQGDVLTA |  | 1660.81641 |
|  |  | QGVVNW |  | 702.35710 |
| H9KMZ3 | Cyclin-dependent kinase 6-like | VPTGDTIFGKIL |  | 1260.71906 |
| H9KTW5 | PREDICTED: glutathione S-transferase-like | HYEENEEIKAAKRK |  | 1744.89768 |
| H9K5H8 | PREDICTED: myophilin | SNKGANQSGINFGNTR |  | 1664.81052 |
| H9K6R4 | PREDICTED: actin, cytoplasmic 1-like, partial | YDESGPSIVH |  | 1103.50060 |
| H9K538 | PREDICTED: hypothetical protein LOC409805 | ALKGTDDIRAPEHTHRPRS |  | 2157.12710 |
|  |  | GTDDIRAPEHTHRPRS |  | 1844.91076 |
|  |  | DMIGRNEPITR |  | 1301.66300 |
| H9KIT9 | PREDICTED: peroxiredoxin-5, mitochondrial | VTVGEKIPTIDL |  | 1284.74096 |
|  |  | VGEKIPTIDL |  | 1084.62504 |
| B0LUE8 | Apolipophorin-III-like protein | NNQLQTAATQKS |  | 1303.66038 |
| H9KMZ2 | PREDICTED: hypothetical protein LOC552453 | ARASAAASEAKANASRN |  | 1645.83599 |
| H9K1E2 | Arginine kinase | GTRGEHTEAEGGIYDISNKR |  | 2190.05366 |
|  |  | HPPKDFGDVDS |  | 1213.54790 |
|  |  | KLSSSDSKSLLK |  | 1292.74220 |
|  |  | VDQAVLDKLETGFSK | N-Acetyl | 1691.88510 |
| H9KKD2 | PREDICTED: hypothetical protein LOC725960 | KAEKPQEEQQEKKAEEKPDRVT |  | 2625.34849 |
| H9K7H5 | PREDICTED: protein takeout-like | TDISNSILKQF |  | 1265.67436 |
| H9KGE6 | PREDICTED: electron transfer flavoprotein subunit beta-like | IAKLKESGHI |  | 1095.65291 |
| Q5XUU6 | Take-out-like carrier protein JHBP-1 | AGGLKSFKIL |  | 1033.64115 |
| H9KR59 | PREDICTED: Na(+)/H(+) exchange regulatory cofactor NHE-RF1-like | DKFDIVQKL |  | 1105.62541 |
| H9KP88 | Aspartate aminotransferase | TPELKPWVL |  | 1082.62512 |
| H9K024 | Tubulin alpha | FSETGAGKHVPRA |  | 1356.70164 |
| H9K626 | PREDICTED: probable citrate synthase 1, mitochondrial-like | AAGMNGLAGPLHG |  | 1165.57785 |
| H9KNB7 | Actin | YDESGPGIVH |  | 1073.49031 |
|  |  | KAGFAGDDAPRAVFPSIVGRPRH |  | 2421.29010 |
|  |  | FPSIVG |  | 619.34500 |
| H9KPL4 | PREDICTED: 60 kDa heat shock protein, mitochondrial-like | KISKGANPVEIRRG |  | 1524.89786 |
|  |  | AKDVRFGAEVRA |  | 1318.72280 |
|  |  | VAVTMGPKGRNVIL |  | 1454.85110 |
| H9K918 | Probably: ATP synthase subunit beta | VVAKAESLAKQ |  | 1143.67363 |
| H9KR70 | PREDICTED: f-box only protein 32-like | REVLLR |  | 785.49942 |
| H9KUC2 | Cuticular protein 12 | HPVGEHLPA |  | 956.49470 |
| H9K3K6 | PREDICTED: apolipoprotein D-like isoform 2 | VEGGGEKPEDV |  | 1115.52170 |
| MULT | Arginine kinases | HPPKDFGDVDS |  | 1213.54790 |
| H9KCL3 | PREDICTED: retinol dehydrogenase 14-like | TVEQGAQTTIHL |  | 1297.67410 |
| H9K639 | PREDICTED: protein disulfide-isomerase | VEDGKIVL |  | 872.50910 |
| H9JYV0 | PREDICTED: 60S ribosomal protein L7 | LPAVPESVLK |  | 1052.63570 |
| H9KEY1 | PREDICTED: ATP synthase subunit O, mitochondrial | pELVKPPIQV | pyroGlu | 1004.61370 |
|  |  | pELVKPPIQVFG | pyroGlu | 1208.70430 |
|  |  | pELVKPPIQVFGIGGRY | pyroGlu | 1754.99570 |
| H9KAU3 | PREDICTED: 10 kDa heat shock protein, mitochondrial-like | AATNAIKRLIP | N-Acetyl | 1209.73150 |
|  |  | AATNAIKRLIPL | N-Acetyl | 1322.81570 |
|  |  | AATNAIKRL | N-Acetyl | 999.59450 |
|  |  | IPLFDRVL |  | 972.58770 |
| H9K748, H9KU35, H9KU34, H9KU33, H9KU31 | PREDICTED: myosin heavy chain, muscle | PKPKPQEGEDPDPTP |  | 1613.78930 |
|  |  | PKPKPQEGEDPDPTPY |  | 1794.85460 |
|  |  | PKPKPQEGEDPDPTPYLF |  | 2055.00560 |
|  |  | DPTPYLF |  | 852.41320 |
|  |  | DPDPTPYLF |  | 1064.49240 |
| H9KUH8, H9KLF7 | PREDICTED: hypothetical protein LOC409090 | VGERGSGAGKGGGGGGSIREAGGSFG |  | 2221.07210 |
| H9KHG2 | PREDICTED: ATP synthase-coupling factor 6, mitochondrial | pEEAKDPIQKL | pyroGlu | 1152.62590 |
| H9KN88 | PREDICTED: probable enoyl-CoA hydratase, mitochondrial | IAEAIKL |  | 757.48250 |
| H9K4S1 | PREDICTED: isochorismatase domain-containing protein 1-like | AVNAAKAVLK | N-Acetyl | 1026.63050 |
| H6CSZ2, H9KBB1, B6VCW8 | Glutathione S-transferase S4 | PVKGLGEPIRF |  | 1212.70970 |
|  |  | FPVKGLGEPIRF |  | 1359.77990 |
| H9K8G6 | PREDICTED: myosin regulatory light chain 2 | APAPINF |  | 729.39320 |
| H9KPS8 | PREDICTED: aldo-keto reductase, NADP+ dependent | AVPTITLNNGHKVPVLG | N-Acetyl | 1772.00630 |
| H9K0L2 | PREDICTED: t-complex protein 1 subunit beta-like isoform 1 | VSLNPVRILK | N-Acetyl | 1180.74050 |
| H9KR11 | PREDICTED: fructose-bisphosphate aldolase-like | VPPAVPGITF |  | 997.57190 |
| H9K9A4 | PREDICTED: 3-hydroxyacyl-CoA dehydrogenase type-2-like | PVDITSTEDVNGAINEIRNKFKKLDVIVNAAGIAVAHK |  | 4060.21550 |

Each protein is represented by accession number, description and a number of peptides supporting it. If identified peptides can be attributed to several proteins all possible accession numbers are listed. Molecular mass, modification and posterior error probability calculated by target-decoy approach are provided for each peptide.
