# Supplementary material for: Protein and Peptide Composition of Male Accessory Glands of Apis mellifera Drones Investigated by Mass Spectrometry
Source: PLoS One. 2015 May 8;10(5):e0125068. doi: 10.1371/journal.pone.0125068 (PMC4425483; doi:10.1371/journal.pone.0125068)
Supplement: S1 Text — (DOCX) [file pone.0125068.s003.docx]

**Supporting Information Text S1. Description of the home-built supporting software.**

**Mass2aa**


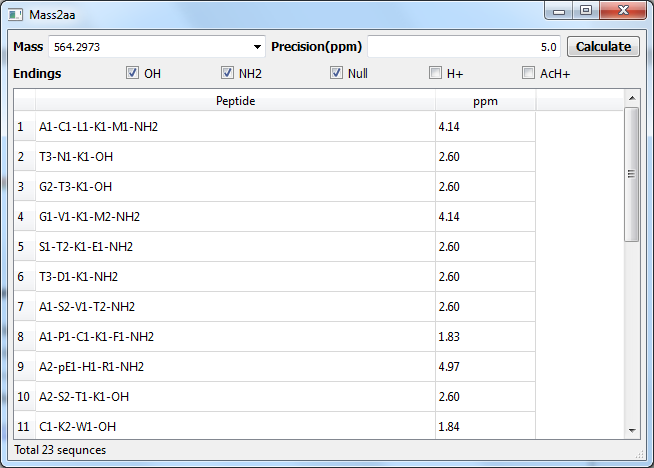


**Figure S1-1. The interface of Mass2aa.**

The interface of Mass2aa is presented at the figure S1-1. The user should provide the mass to search compositions for (**Mass**), desired mass accuracy in ppm for the match (**Precision(ppm)**) and type of peptide structure to consider (**Endings**).

Ending types:

OH – peptide with free acid C-terminus;

NH2 – peptide with amidated C-terminus;

Null – bare amino acid combination;

H+ – b-ion;

AcH+ – b-ion with acetylated N-terminus

After the calculation is completed, the list of possible amino acid compositions (**Peptide**) and corresponding mass error in ppm (**ppm**) is shown to the user, the total number of possible compositions is displayed in the footer. All results computed in the current session are stored in memory and can be redisplayed instantly by selecting the corresponding mass from drop-down field.

The compositions are built using 19 standard amino acids (leucine is indistinguishable from isoleucine) extended with hydroxyproline, pyroglutamate, oxidized methionine and carboxamidomethylated cysteine. The possibility of disulfide bridge formation is included in the calculation.

**Fuzzymatch**


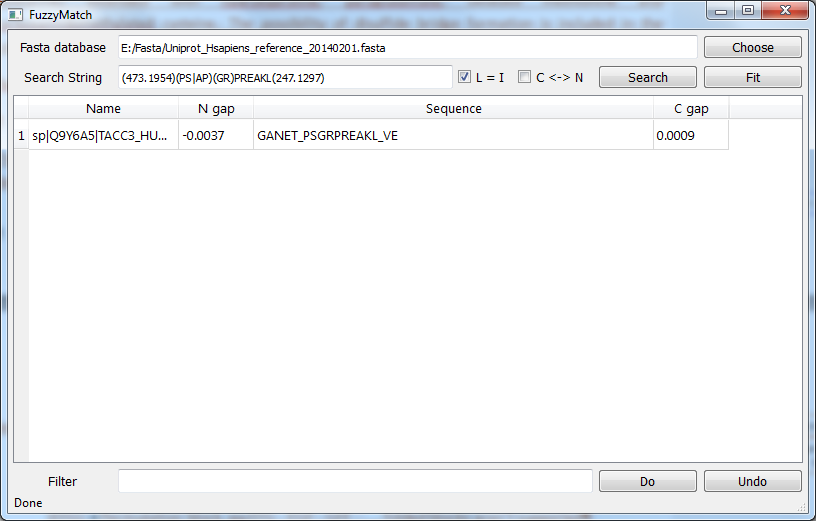


**Figure S1-2. The interface of Fuzzymatch.**

The interface of Fuzzymatch is presented in figure S1-2. The user should select the protein database in FASTA format (**Fasta database**) to perform the search, search pattern (**Search String**) in specific format.

Search pattern rules:

(####)ABCD(EFG)HI($$$$)

####: Mass block, N-terminal part (float number or nothing)

ABCD: AA sequence with single letter codes

(EFG): Permutation block, e.g. EFG - EGF - GFE - ..., folded blocks aren't supported

(ABC|EFG): Alternate sequence, e.g. (ABC) or (EFG)

HI: AA sequence with single letter codes

($$$$): Mass block, C-terminal part (float number or nothing)

Examples of valid search patterns:

QWERTY

QWERTY(IP)

(123.45)QWERTY

QWETY(123.45)

(123.45)QWE(RTY)(IP|AS)(456.78)

Check boxes allow to perform the search with leucine indistinguishable from isoleucine (**L = I**) and to search in both directions (**C <-> N**), the default setting is to consider that search string is written from N-terminal to C-terminal. **Search** button performs the search using the search string, while **Fit** button performs the search with the same string assuming possible amino acid substitutions. All matches with the smallest number of substitutions from the search string are displayed (the second option is much slower and not scheduled by default).

Upon the completion all matches found in the database are listed in the table, each protein is indicated by the header from the fasta file. Each match is designated by the sequence matched for the core of the search string (amino acid sequence with all permutation or alternative blocks) separated with underscore “**_**” from the best matches for mass blocks. **N gap** and **C gap** columns contain the difference in Da between the mass of the mass block and mass of the amino acids in the corresponding match.

Filter window is located under the results table. It is used to filter out some of the matches in the case when their number is large; the format of filter string is the same as for search string.
