# Supplementary material for: Protein and Peptide Composition of Male Accessory Glands of Apis mellifera Drones Investigated by Mass Spectrometry
Source: PLoS One. 2015 May 8;10(5):e0125068. doi: 10.1371/journal.pone.0125068 (PMC4425483; doi:10.1371/journal.pone.0125068)
Supplement: S2 Text — (DOCX) [file pone.0125068.s004.docx]

**Supporting Information Text S2. Comparison of SEQUEST and *de novo* results for the same peptide.**

Figure S2-1 shows one of the poorly identified peptides, SEQUEST proposes the following sequence for it: QQDQYNSDQRPIHR-OH. However, it is easy to see that only few peaks, excluding the most intense ones, get annotated, moreover the number of ions rationalized is quite low (see table S2-1).

**Figure S2-1. Fragmentation spectra of the peptide (m/z 892.9249) with highlighted matched ions as displayed by Thermo Proteome Discoverer 1.3.**

**Table S2-1. Ions identified by SEQUEST for the peptide with m/z 892.9249.**

| b | Error (ppm) | AA | Error (ppm) | y |
| --- | --- | --- | --- | --- |
| 1 | - | Q |  | 14 |
| 2 | - | Q | - | 13 |
| 3 | - | D | - | 12 |
| 4 | - | Q | - | 11 |
| 5 | - | Y | - | 10 |
| 6 | - | N | - | 9 |
| 7 | - | S | - | 8 |
| 8 | +5.5 | D | -18.2 | 7 |
| 9 | +1.7 | Q | +2.3 | 6 |
| 10 | - | R | -0.1 | 5 |
| 11 | +3.2 | P | - | 4 |
| 12 | - | I | +0.7 | 3 |
| 13 | - | H | - | 2 |
| 14 |  | R | - | 1 |

Using the *de novo* approach, the following sequence was obtained for the spectrum: AAHPEEDDGGQPRPPGR-NH_2_; this sequence helps to explain all intense peaks, and the number of annotated ions is significantly larger (Fig. S2-2). It should be mentioned as well, that mass accuracy for the identified fragment peaks improves significantly (compare Table S2-1 and Table S2-2).


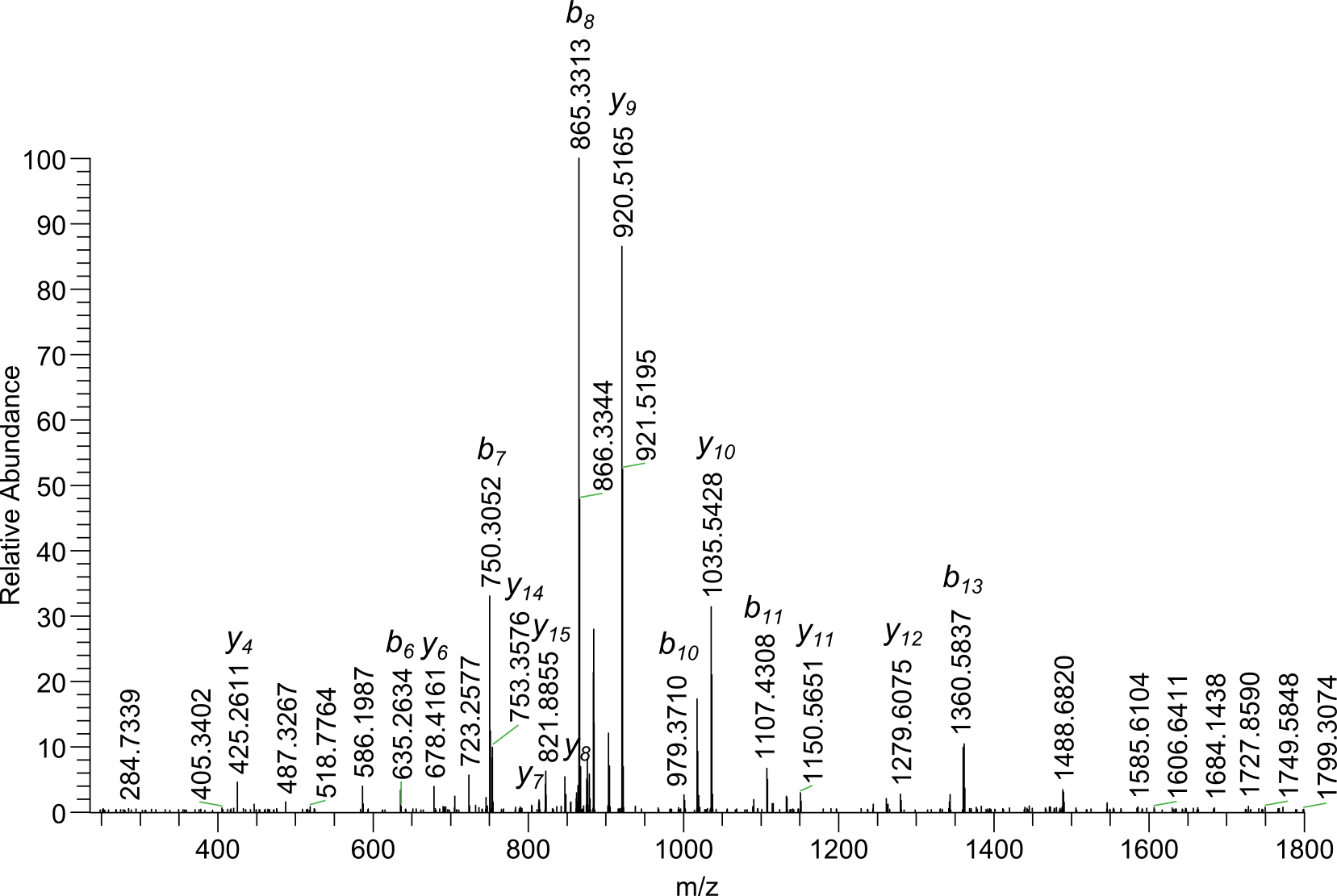


**Figure S2-2.** **Fragmentation spectra of the peptide (m/z 892.9249) with ions annotated during *de novo* sequencing.**

**Table S2-2. Ions identified by *de novo* sequencing for the peptide with m/z 892.9249.**

| b | Error (ppm) | AA | Error (ppm) | y |
| --- | --- | --- | --- | --- |
| 1 |  | A |  | 17 |
| 2 |  | A |  | 16 |
| 3 |  | H | -2.6 | 15 |
| 4 |  | P | -0.8 | 14 |
| 5 |  | E |  | 13 |
| 6 | 0.1 | E | -4.9 | 12 |
| 7 | -0.3 | D | -7.9 | 11 |
| 8 | -1.2 | D | -2.0 | 10 |
| 9 |  | G | -0.7 | 9 |
| 10 | -4.3 | G | -1.6 | 8 |
| 11 | -1.9 | Q | 2.4 | 7 |
| 12 |  | P | -0.1 | 6 |
| 13 | -2.9 | R |  | 5 |
| 14 |  | P | -1.3 | 4 |
| 15 |  | P |  | 3 |
| 16 | 2.3 | G |  | 2 |
| 17 |  | R |  | 1 |
