# Supplementary material for: Protein and Peptide Composition of Male Accessory Glands of Apis mellifera Drones Investigated by Mass Spectrometry
Source: PLoS One. 2015 May 8;10(5):e0125068. doi: 10.1371/journal.pone.0125068 (PMC4425483; doi:10.1371/journal.pone.0125068)
Supplement: S3 Text — (DOCX) [file pone.0125068.s005.docx]

**Supporting Information Text S4. Examples of *de novo* sequencing.**


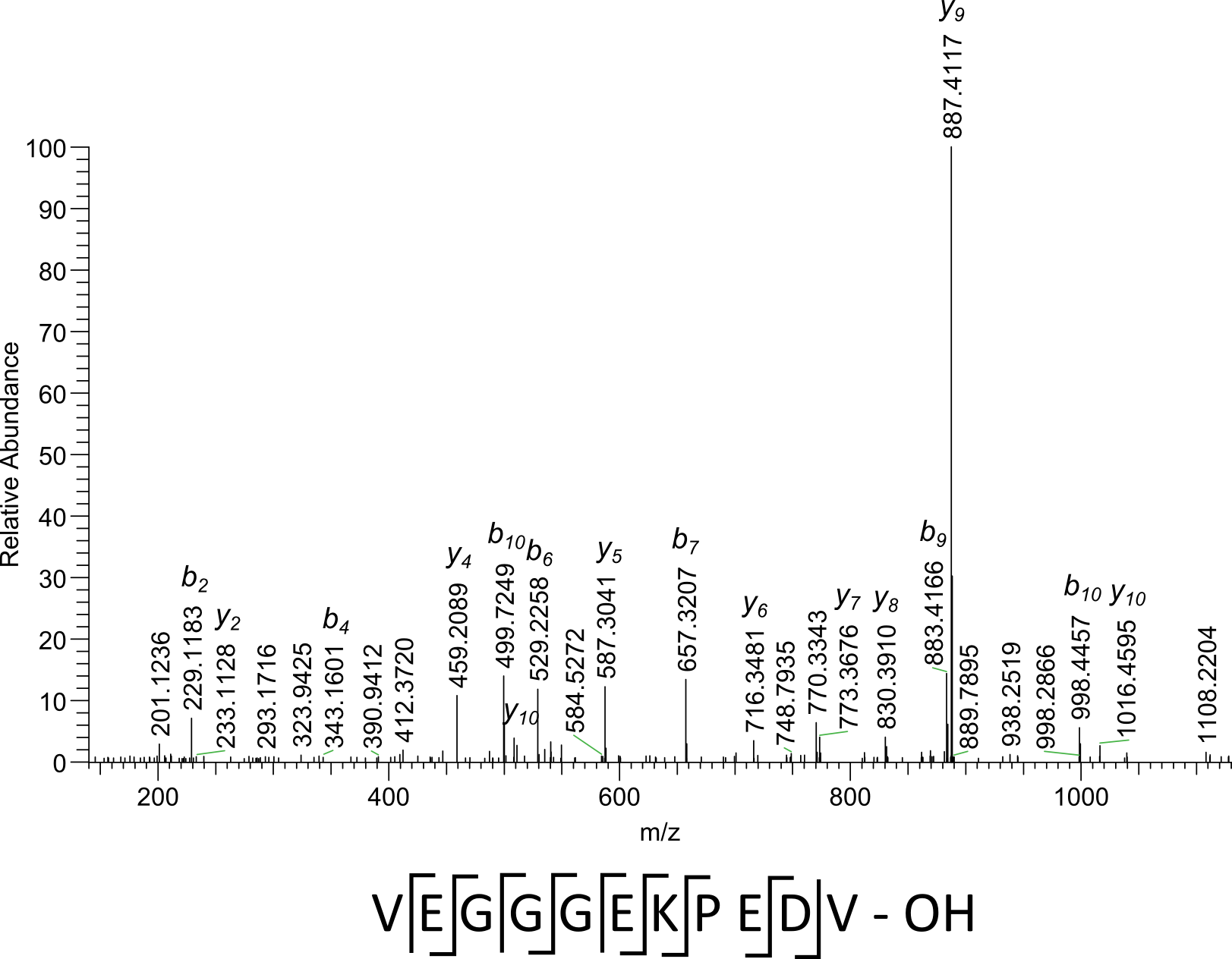


**Fig. S4-1. The fragmentation spectrum of a peptide with accurate measured mass of 1114.5144 Da (M), doubly-charged precursor (m/z 558.2645). The sequence under the spectrum shows observed cleavages.**

The main “quality control” principle of *de novo* sequencing results is the same like for SEQUEST ones: all intense peaks should be explained by the proposed sequence. In the case of a peptide with accurate measured mass of 1114.5144 Da (Fig. S4-1) a prominent row of y-type ions can be identified, most of them have also complementary b-type ions. The mass accuracy of all annotated peaks is less than 10 ppm (typically less than 2). The gap between 7^th^ and 10^th^ amino acids can be explained according to exact mass as a combination of proline and glutamate or as two hydroxyprolines (error – 3.29 ppm). According to known fragmentation rules, the most probable sequence is PE (proline rule). The sequence hit VEGGGEK(PE|PP)DV, where (PE|PP) stands for all possible combinations of PE pair, or PP pair, was given to Fuzzymatch software. The only possible match is the fragment of apolipoprotein D-like isoform 2 protein, having the sequence VEGGGEKPEDV-OH (accuracy 0.18 ppm). This database hit perfectly matches the established sequence, including the position of the PE pair.


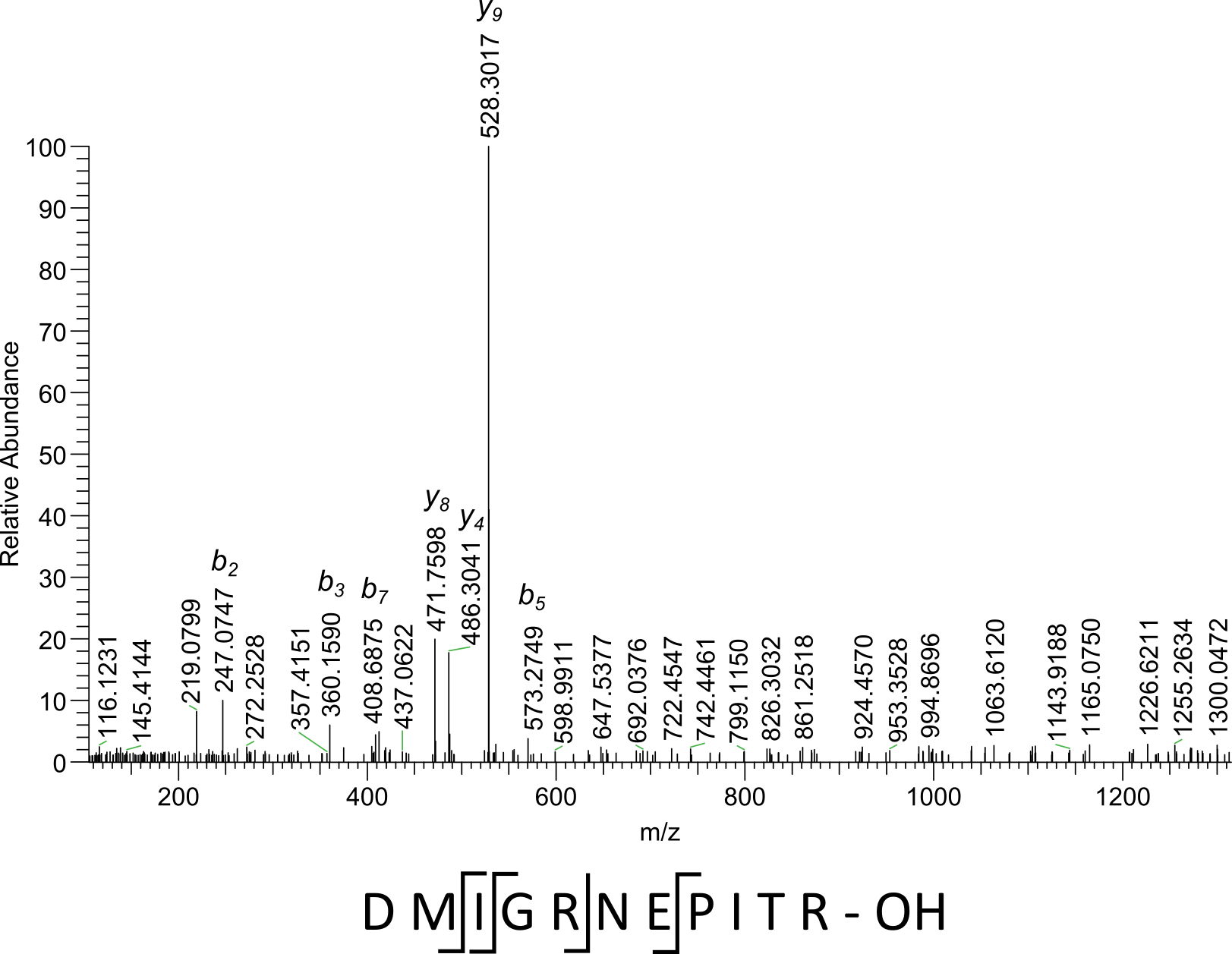


**Fig. S4-2. The fragmentation spectrum of a peptide with accurate measured mass of 1300.6557 Da (M), triply-charged precursor (m/z 434.5592). The sequence under the spectrum shows observed cleavages.**

The fragmentation of the peptide with accurate measured mass of 1300.6557 Da (Fig. S4-2) is quite inefficient, there are only few signals in the mass spectrum. Using the accurate mass it is possible to find the composition of b_2_ – the only variant is DM (0.02 ppm), for y_4_ (mass 486.3041) there are 25 possible amino acid compositions, including free acid and amide C-terminal, for the gap between b_3_ and b_7_ (456.2087) there are 22 possible compositions. Using database matching query (DM)L(942.5128) only one hit was found, that has a possible structure for y_4_ (1.27 ppm accuracy for C-terminal block). Its sequence is DMIGRNEPITR-OH, this is the fragment of hypothetical protein LOC409805. Weak noise-level peak corresponding to b_5_ and several peaks of internal ions give additional evidence for the proposed structure. The mass accuracy for the complete peptide is 0.08 ppm.


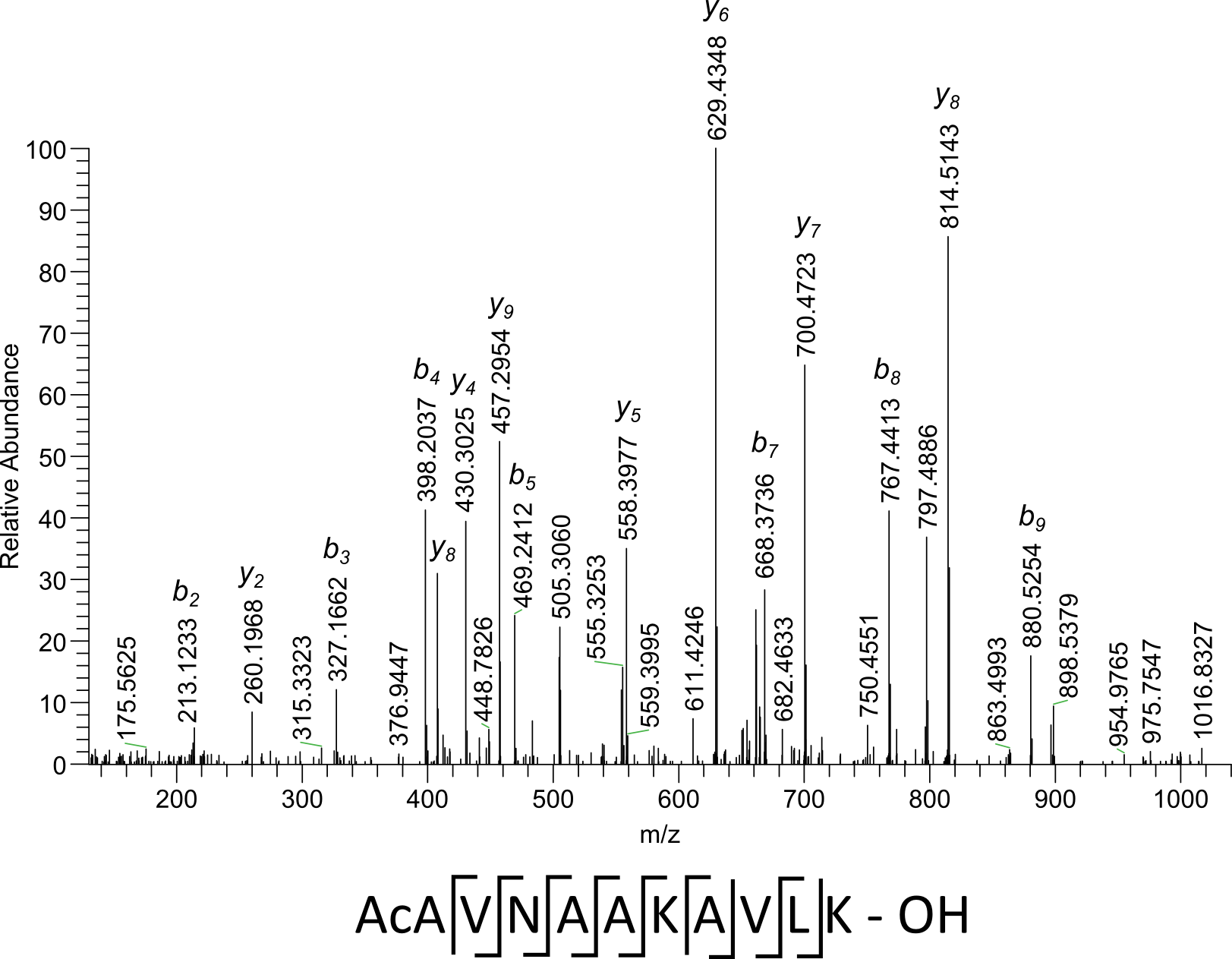


**Fig. S4-3. The fragmentation spectrum of a peptide with accurate measured mass of 1025.6232 Da (M), doubly-charged precursor (m/z 513.8189). The sequence under the spectrum shows observed cleavages.**

The fragmentation pattern on figure S4-3 provides intense series of b- and y-ions, which help establishing the complete peptide structure. The first amino acid residue in the sequence has a mass of 113.0470 Da, which can be treated as isomeric AcA (acetylated alanine) or Hyp (hydroxyproline). The first possibility fits better to database sequences, the identified peptide is related to isochorismatase domain-containing protein 1-like protein. Mass accuracy – 0.10 ppm.


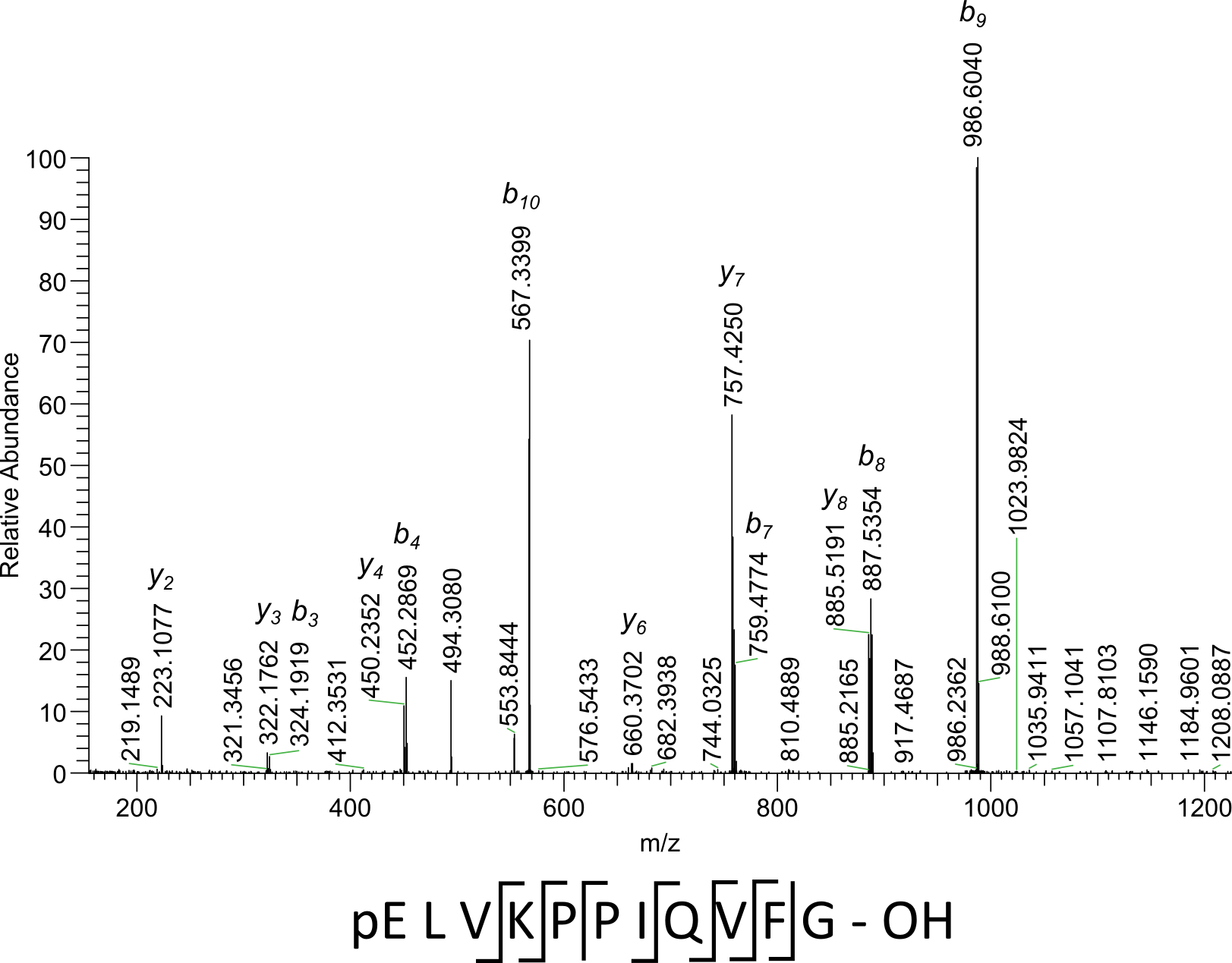


**Fig. S4-4. The fragmentation spectrum of a peptide with accurate measured mass of 1207.6968 Da (M), doubly-charged precursor (m/z 604.8557). The sequence under the spectrum shows observed cleavages.**

The spectrum (Fig. S4-4) is dominated by strong b-type ions and usually less intense complementary y-ions. The sequence established for this spectrum is provided under the picture. There are two gaps in it – the first one is 210.1349 Da in size. According to the mass, it could be only a PL combination. The second one is located at the beginning of peptide. There are two possibilities for the structure of b_3_-ions (m/z 324.1919): pELV or PHypL. The best fit found in the database has the sequence QLVKPPIQVFG-OH and represents an ATP synthase subunit O. It is well known that pyroglutamate is formed post-translationally from glutamine. Thus, the proposed sequence is quite reasonable. Mass accuracy for the complete peptide is 0.25 ppm.
